# Anti-Inflammatory Secondary Metabolites from *Penicillium* sp. NX-S-6

**DOI:** 10.3390/md23070280

**Published:** 2025-07-04

**Authors:** Hanyang Peng, Jiawen Sun, Rui Zhang, Yuxuan Qiu, Yu Hong, Fengjuan Zhou, Chang Wang, Yang Hu, Xiachang Wang

**Affiliations:** 1 Jiangsu Key Laboratory for Functional Substances of Chinese Medicine, Nanjing University of Chinese Medicine, Nanjing 210023, China; 17379935106@163.com (H.P.); 20230867@njucm.edu.cn (J.S.); 20220952@njucm.edu.cn (R.Z.); 13901591202@163.com (Y.Q.); 15105186189@163.com (Y.H.); zhoufengjuan2014@outlook.com (F.Z.); wangchang@njucm.edu.cn (C.W.); 2 Fujian Province Key Laboratory for the Development of Bioactive Material from Marine Algae, College of Oceanology and Food Science, Quanzhou Normal University, Quanzhou 362000, China

**Keywords:** sorbicillinoid, indolinone alkaloid, steroid, *Penicillium* sp., anti-inflammatory

## Abstract

Five new natural products, including two sorbicillinoids (**1**–**2**), one indolinone alkaloid (**10**), one tetracyclic steroid (**11**), and one α-pyrone derivative (**14**), were identified from the endophytic *Penicillium* sp. NX-S-6, together with thirteen known natural products. The structures of new compounds were unambiguously elucidated by comprehensive spectroscopic analyses (NMR, MS), as well as electronic circular dichroism (ECD) calculation. Notably, quinosorbicillinol (**1**) was identified as a rare hybrid sorbicillinoid incorporating a quinolone moiety, representing a unique structural scaffold in this natural product class. Biological evaluation revealed that Compounds **1**, **4** and **8** potently inhibited the production of nitric oxide and interleukin 6 in lipopolysaccharide (LPS)-stimulated RAW264.7 macrophages. Mechanistic studies furthermore demonstrated that Compounds **4** and **8** effectively suppressed interleukin-1β secretion in LPS-induced immortalized mouse bone marrow-derived macrophages (iBMDMs) by blocking NLRP3 inflammasome activation. This inhibition was attributed to their ability to disrupt the assembly of the NLRP3-caspase-1 complex, a key event in the pathogenesis of inflammatory disorders. These findings not only expand the structural diversity of endophyte-derived natural products but also highlight their potential as lead compounds for developing anti-inflammatory therapeutics targeting the NLRP3 pathway.

## 1. Introduction

Marine natural products denote organic metabolites derived from marine organisms, encompassing sponges, algae, bryozoans, cnidarians, tunicates, echinoderms, microorganisms, mangroves and other intertidal flora [1,2]. These metabolites include diverse organic classes such as polyketides, peptides, terpenes, sterols, alkaloids, etc. [1,3,4,5]. Research in this field involves not only the isolation, purification and structure elucidation, but also the bioactivity profiling [3,6] and biosynthetic pathway dissection [7,8]. Sorbicillinoids, a distinct family of polyketides characterized by a sorbyl side chain, are predominantly isolated from marine fungi [9,10,11,12]. Their structural uniqueness lies in the diversity of monomeric, dimeric, trimeric architectures, and even hybrid scaffolds incorporating heterocyclic moieties (e.g., quiniline, indole) [9,13,14]. Since the first sorbicillinoid was reported in 1948 [15], over 170 sorbicillinoid congeners have been identified, most of which exhibit significant biological activities—including anti-inflammatory, anticancer, antimicrobial and antioxidant properties [9,10,13,16,17]. These attributes position them as promising lead compounds for pharmaceutical and agrochemical development, particularly due to their selective targeting of disease-relevant molecular pathways [9,10].

In the pursuit of novel bioactive natural products from microorganisms inhabiting niche ecological niches [18,19,20], the endophytic *Penicillium* sp. NX-S-6 was identified as a promising isolate based on its demonstrated ability to biosynthesize unique aminosorbicillinol derivatives [20]. To further expand the structural diversity of the sorbicillinoid natural product class, the “one strain many compounds” (OSMAC) approach was systematically employed by using solid rice, potato dextrose and corn meal. This strategy involves the manipulation of cultivation parameters, including nutrient composition, temperature, and aeration, to induce the production of cryptic secondary metabolites that are not expressed under standard fermentation conditions. Upon comprehensive analysis of high-resolution liquid chromatography–mass spectrometry (HR-LC-MS) data, a molecular ion with a protonated adduct [M + H]^+^ at *m*/*z* 407.2 was detected. The ultraviolet-visible (UV-Vis) spectral characteristics of this ion exhibited a distinctive absorption profile reminiscent of sorbicillinoids; however, the observed molecular mass was lower than that typically associated with dimeric sorbicillinoid congeners, suggesting the presence of a hitherto unreported structural architecture.

**Figure 1:**
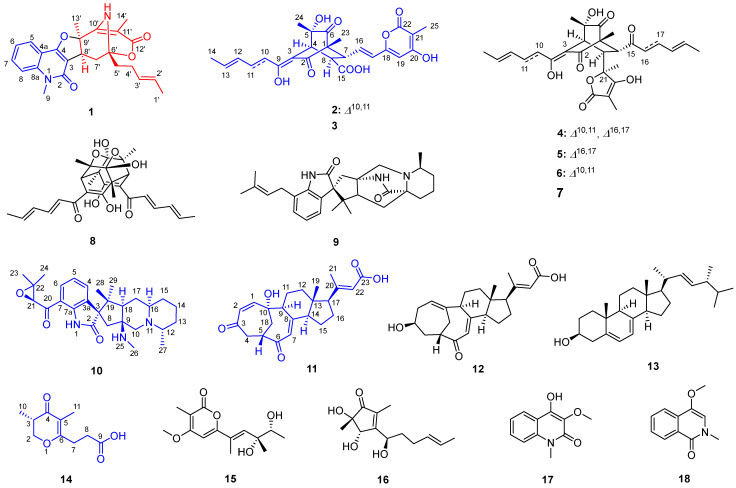
Structures of Compounds **1**–**15** from *Penicillium* sp. NX-S-6.

To facilitate structural elucidation, a large-scale fermentation (30 L) was conducted using solid rice medium, a proven substrate for enhancing the production of diverse secondary metabolites in filamentous fungi due to its rich nutrient composition and favorable physical properties. Following fermentation, the crude extract was subjected to a multi-step purification process. The purified compound was subsequently characterized using a suite of advanced spectroscopic techniques, including one- and two-dimensional nuclear magnetic resonance (1D/2D NMR) spectroscopy, high-resolution electrospray ionization mass spectrometry (HR-ESI-MS), and electronic circular dichroism (ECD) analysis. These studies revealed the compound to be a hybrid sorbicillinoid incorporating an anthranilic acid moiety, featuring a novel 6/6/5/6/6 fused pentacyclic carbon framework (**1**). In addition to this novel metabolite, four additional new natural products and thirteen known compounds were successfully isolated and identified (Figure 1). This report details the fermentation protocols, isolation procedures, structural elucidation strategies, and anti-inflammatory bioactivity evaluations of these natural products.

## 2. Results and Discussion

Preliminary LC-MS analyses of crude extract prepared from *Penicillium* sp. NX-S-6 culture revealed a set of potentially new secondary metabolites. Scale-up fermentation (30 L) of this strain, followed by extraction and chromatographic purification, yielded five new compounds: quinosorbicillinol (**1**, yield: 0.04 mg/L), bisorbicillpyrone B (**2**, yield: 0.14 mg/L), citrinadin E (**10**, yield: 0.32 mg/L), norcyclocitrinoic acid C (**11**, yield: 0.12 mg/L), stapyrone I (**14**, yield: 0.12 mg/L).

**Figure 2:**
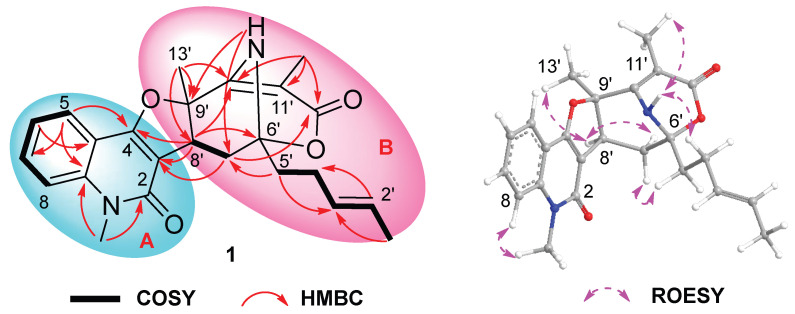
^1^H−^1^H COSY, key HMBC and ROESY correlations of Compound **1**.

Quinosorbicillinol (**1**) was isolated as a yellow powder. The molecular formula was decided as C_24_H_26_N_2_O_4_ from HRESIMS peak at *m*/*z* 405.1819 [M − H]^−^ (calcd for C_24_H_25_N_2_O_4_, 405.1814), indicating 13 degrees of unsaturation. The ^1^H NMR spectrum of **1** (Table 1) showed signals attributable to four aromatic protons [*δ*_H_ 7.23 (t), 7.34 (d), 7.59 (t), 7.84 (d)], two olefinic protons [*δ*_H_ 5.58 (m), 5.65 (m)], one methine group [*δ*_H_ 3.84 (brs)], one N-methyl group [*δ*_H_ 3.70 (s)], three methylene groups [*δ*_H_ 2.08 (^1^H, m), 2.17 (^2^H, t), 2.41 (^2^H, m), 2.62 (^1^H, dd)], and three methyl groups [*δ*_H_ 1.51 (s), 1.73 (d), 1.75 (s)]. The ^13^C NMR spectrum (Table 1) showed total of 24 carbon signals, which can be assigned to two carbonyls (*δ*_C_ 162.3, 173.5), six sp^2^ quaternary carbons (*δ*_C_ 101.2, 105.3, 114.9, 139.6, 155.9, 165.6), six sp^2^ methines (*δ*_C_ 114.5, 121.8, 122.7, 127.0, 129.9, 131.3), one N-methyl (*δ*_C_ 29.9), one methine (*δ*_C_ 34.7), three methylene (*δ*_C_ 26.9, 31.1, 39.4), three methyl (*δ*_C_ 6.5, 18.1, 26.0) and two oxygenated quaternary carbons (*δ*_C_ 82.0, 89.2), as revealed by HSQC spectrum. Further detailed analysis of 2D NMR data allowed the construction of two substructures, as shown in Figure 2. The ^1^H-^1^H COSY spectra revealed the existence of an ortho-substituted phenyl ring according to the main spin system from H-5 to H-8, which shown at 7.84 (d, *J* = 7.9 Hz, H-5), 7.23 (t, *J* = 7.6 Hz, H-6), 7.59 (t, *J* = 7.7 Hz, H-7) and 7.34 (d, *J* = 8.5 Hz, H-8). The key HMBC correlations from N-methyl (CH_3_-9) to C-2 and C-8a, and from H-5 to C-4, and C-8a, supported the construction of the quinolone structure (subunit A in Figure 2), which turned out to be 4-hydroxy-3-methoxy-1-methyl-2(1*H*)-quinolone (**17**) [21] with different C-3 substitution. The ^1^H-^1^H COSY correlations from H-1′ to H-5′ indicated the characteristic sorbyl side chain of sorbicillinoid, while the hexatomic ring varied greatly. Firstly, an α,β-unsaturated ketone can be readily recognized from the ^13^C NMR of C-10′ (*δ*_C_ 165.6), C-11′ (*δ*_C_ 101.2) and C-12′ (*δ*_C_ 173.5). And a methyl was located at C-11′ through the HMBC correlations from H_3_-11′ to C-10′, C-11′and C-12′. Another methyl at *δ*_H_ 1.75 (s) showed HMBC correlations to C-8′ (*δ*_C_ 34.7), C-9′ (*δ*_C_ 82.0) and C-10′, indicating the C-9′ with a methyl substitution. The ^1^H-^1^H COSY cross peaks of H_2_-7′ (*δ*_H_ 2.08 and 2.62)/H-8′ (*δ*_H_ 3.84), together with the HMBC correlations from H-8′ to C-6′ (*δ*_C_ 89.2) and C-10′, and from H-5′ (*δ*_H_ 2.17) to C-7′ (*δ*_C_ 31.1), and from H_2_-7′ to C-12′ (*δ*_C_ 173.5), revealed the existence of a 10-membered macrocyclic lactone. Moreover, an amino proton at *δ*_H_ 4.94 displayed strong HMBC correlation peaks C-7′ and C-9′, which helped to construct an amino bridge between C-6′ and C-9′. The quinolone and variational sorbicillinoid were hybridized through a dihydrofuran ring, elucidated through key HMBC correlations from H_2_-7′ to C-2, and from H-8′ to C-2 and C-4 (Figure 2). Thus, the planar structure of **1** was elucidated as shown in Figure 1.

**Figure 3:**
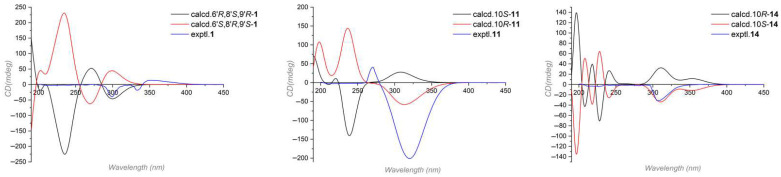
The experimental and calculated ECD spectra of Compounds **1**, **11** and **14**.

Compound **1**′s relative configuration was established by the ROESY spectrum. ROESY correlations of H-8′/H-13′ and H-7′α/H-8′ indicated they were co-facial and arbitrarily fixed in α-orientation. The NOE cross peaks of H-4′/N-H and N-H/H_3_-11′ indicated the nitrogen bridge was β-oriented (Figure 2). Several attempts to crystallize **1** did not produce material suitable for X-ray experiments. Alternatively, electronic circular dichroism (ECD) analysis was used for absolute configuration determination [22,23]. Specifically, time-dependent density functional theory (TDDFT) was used to calculate the ECD spectra of the two possible enantiomers of **1**. And the theoretical ECD spectra were then compared to the actual ECD spectrum. The better match of the 6′*R*, 8′*S*, 9′*R* isomer in the ECD spectrum (Figure 3) helped to decide the absolute configuration of **1** as 6′*R*, 8′*S*, 9′*R*. Based on the above analysis, Compound **1** was identified as a new hybrid sorbicillinoid with a quinolone moiety, and it was named quinosorbicillinol.

From a biosynthetic perspective, quinosorbicillinol was constructed from two distinct precursors—a sorbicillinoid and a quinolone—derived from separate pathways. Although both precursor types are widely distributed in fungi [9,24], no examples of their biosynthetic pathway hybridization have been reported to date. The discovery of quinosorbicillinol has highlighted nature’s astonishing ability to synthesize unexpected natural products. Here, we proposed a hypothetical biosynthetic pathway for quinosorbicillinol (Scheme S1). Initially, the sorbicillinoid precursor was generated by iterative polyketide synthases. An aminotransferase then replaced the C-10 ketone group with an amino group. Nucleophilic attack by this amino group on the C-6 ketone led to the formation of the first nitrogen-containing six-membered heterocyclic ring. Subsequently, the highly nucleophilic C-3 position of 4-hydroxy-N-methyl-2-quinolone attacked the C-8 ketone group of the polyketide chain to form a C-C bond. A furan ring was then constructed through dehydration and cyclization. Finally, the product was released as an ester, and a putative oxidation step yielded the final compound, quinosorbicillinol (**1**).

**Figure 4:**
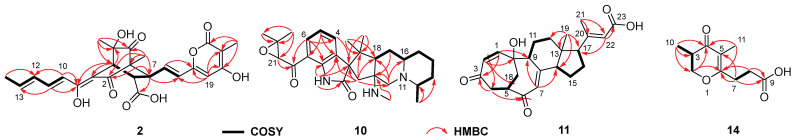
^1^H−^1^H COSY and key HMBC correlations of Compounds **2**, **10**, **11** and **14**.

**Figure 5:**
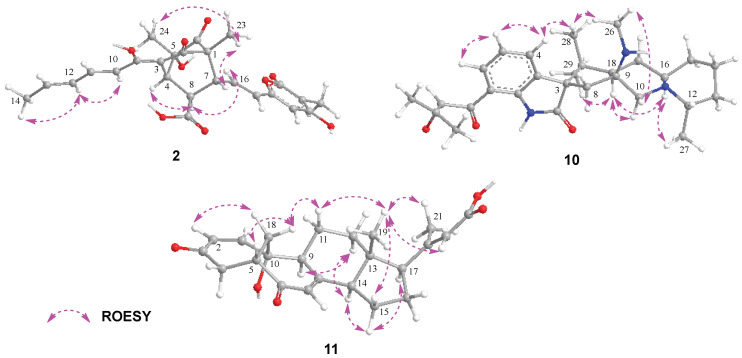
Key ROESY correlations of Compounds **2**, **10** and **11**.

The molecular formula C_25_H_26_O_9_ of Bisorbicillpyrone B (**2**) was determined by HRESIMS at *m*/*z* 469.1497 [M − H]^−^ (calcd for C_25_H_25_O_9_, 469.1499), indicating 13 degrees of unsaturation. The ^13^C NMR data (Table 2) showed the presence of 25 carbon signals, which can be sorted into four methyls, three sp^2^ methines, twelve olefinic carbons, two quaternary carbons, two carboxyl and two ketones, with the aid of the HSQC experiment. The existence of a sorbyl side chain from H-10 to H_3_-14 in ^1^H NMR and ^1^H-^1^H COSY spectra indicated that 2 was a sorbicillinoid congener (Figure 2). Its NMR data closely resembled those of bisorbicillpyrone A (**3**) [25], with structural divergence about an extra double bond. Specifically, the key ^1^H-^1^H COSY correlations from H-10 to H_3_-14, along with related key HMBC correlations, helped to assign the *Δ*^10,11^ at **2** (Figure 4). The relative configuration of **2** was established by ROESY correlations. Compared to bisorbicillpyrone A (**3**) [25], the similar NOE correlations of H-4/H-8 and H-8/H-16 indicated they were *α*-oriented. On the other hand, the key ROESY cross-peaks of H-7/H_3_-23 and H_3_-23/H_3_-24 revealed that they were in β-orientation. The double bonds of *Δ*^10,11^ and *Δ*^16,17^ were both in *E*-geometry format from the coupling constant of H-11 (*δ*_H_ 7.34, dd, *J* = 14.9, 15.0) and H-17 (*δ*_H_ 6.18, d, *J* = 15.3). The *Δ*^12,13^ was also in *E*-geometry based on the ROESY correlation of H-2/H_3_-14 (Figure 5). Thus, thorough analyses of 1D and 2D NMR spectra cumulatively established the structure of Compound **2**, as depicted in Figure 1, and it was subsequently named bisorbicillpyrone B.

Citrinadin E (**10**) was isolated as a pale yellow oil; the formula C_28_H_39_N_3_O_3_ was determined by HRESIMS at *m*/*z* 466.3070 [M + H]^+^ (calcd for C_28_H_40_N_3_O_3_, 466.3069), indicating 11 degrees of unsaturation. Its ^1^H and ^13^C NMR data (Table 2) closely resembled those of citrinadin B [26,27]. The obvious difference was the absence of an oxygenated quaternary carbon (*δ*_C_ 82.4) in **10**, and the HRESIMS data revealed that **10** lacked one oxygen atom compared to citrinadin B, which indicated the deficiency of a hydroxyl group at C-18. This was confirmed by the key HMBC correlations from H-28 and H-29 to C-3, C-18 and C-19 (Figure 4). Thus, the planar structure of **10** was established, as shown in Figure 1. Recently, the stereochemical structure of citrinadin B has been revised by total synthesis [27]. The relative configuration at the stereocenters of **10** was indirectly established through the analyses of ROESY correlations (Figure 4) and their comparison with those of citrinadin B [27]. The key ROESY correlations of H-16/H-18, H-16/H_3_-27, H-10α/H-18, and H-18/H_3_-29 revealed that they were on the same side of the molecule as citrinadin B and analysis of the ROESY spectrum. On the other hand, H-4, Me-26 and Me-28 were on the opposite side (Figure 5). Furthermore, the highly consistent ^1^H and ^13^C NMR data at C-7 side chain indicated the configuration at C-21 could be assigned as 21S*. Therefore, all the results were consistent with the chemical structure of **10**, as displayed in Figure 1.

Compound **11** was isolated as a colorless powder. HRESIMS analysis of **11** revealed the molecular formula C_23_H_28_O_5_, requiring 10 degrees of unsaturation. Analyzing its ^1^H and ^13^C NMR data (Table 2) revealed the structural similarity with norcyclocitrinoic acid A [28], the nearly isolated steroid featuring an unusual 7/7/6/5-tetracyclic scaffold. The main difference was an extra keto carbonyl carbon and an oxygenated quaternary carbon observed in **11**. By analysis of its 2D NMR, Compound **11** was demonstrated to have a ketone carbonyl group at C-3 instead of a hydroxyl group in norcyclocitrinoic acid A. Moreover, the double bond was shifted from positions *Δ*^1,10^ to *Δ*^1,2^, and C-10 was substituted with a hydroxyl. This was confirmed by the ^1^H-^1^H COSY correlation of H-1/H-2, and HMBC correlations from H-1 to C-3 and from H-2 to C-10. Based on the ROESY spectrum and their comparable NMR chemical shifts, the relative configuration of **11** was determined to be similar as that of norcyclocitrinoic acid A with ROESY correlations of H-9/H-11α, H-11α/H-14, H-14/H-17, H-14/H-15α, H-15α/H-17, H-15β/H_3_-19, H-11β/H_3_-19, H_3_-19/H_3_-21, H-11β/H_b_-18, and H_b_-18/H-5 (Figure 5). According to the ECD calculation result, the C-10’s absolute configuration of **11** was determined as 1*0R* with an experimental ECD curve more consistent with the calculated curve (Figure 3). As a result, **11** was elucidated as a new steroid with a 7/7/6/5-tetracyclic system and given the designation norcyclocitrinoic acid C.

Stapyrone I (**14**) was obtained as a pale yellow oil. Based on the HRESIMS ion peak at *m*/*z* 197.0818 [M − H]^−^, **14** had a molecular formula of C_10_H_14_O_4_ with 4 degrees of unsaturation. Comparison of the NMR data (Table 3) disclosed high similarity with stapyrone G [29], with the principal differences being the replacement of the methyl formate group (δ_C_ 172.9) in stapyrone G with a carboxylic acid in **14** (δ_C_ 177.2), indicated by diagnostic HMBC correlations of H-7/C-9 and H-8/C-9. The absolute configuration of **14** was determined as 10*S* based on the ECD calculations (Figure 3).

Thirteen known compounds with different structure styles were also isolated and identified as bisorbicillpyrone A (**3**) [25], trichotetronine (**4**) [30], dihydrotrichotetronine (**5**), 10,11-dihydrobislongiquinolide (**6**) [31], 10,11,16,17-tetrahydrobislongiquinolide (**7**) [31], trichodimerol (**8**) [32,33], chrysogenamide A (**9**) [34], norcyclocitrinoic acid A (**12**) [28], ergosterol (**13**) [20], stapyrone B (**15**) [29], 5-hydroxycyclopenicillone (**16**) [35], 4-hydroxy-3-methoxy-1-methyl-2(1*H*)-quinolone (**17**) [21], and 4-methoxy-2-methylisoquinolin-1(2*H*)-one (**18**) [36].

The anti-inflammatory activities of all yield natural products were evaluated by using routine protocols [37]. All tested compounds showed no cytotoxicity to macrophages at concentrations of 50 μM. Notably, quinosorbicillinol (**1**), trichotetronine (**4**) and trichodimerol (**8**) showed considerable inhibitory efficacy on both the production of nitric oxide and IL-6 in LPS-stimulated RAW264.7 cells. Meanwhile, bisorbicillpyrone B (**2**), 10,11-dihydrobislongiquinolide (**6**), chrysogenamide A (**9**) and norcyclocitrinoic acid C (**11**) showed weak inhibition on NO release (Table 4).

**Figure 6:**
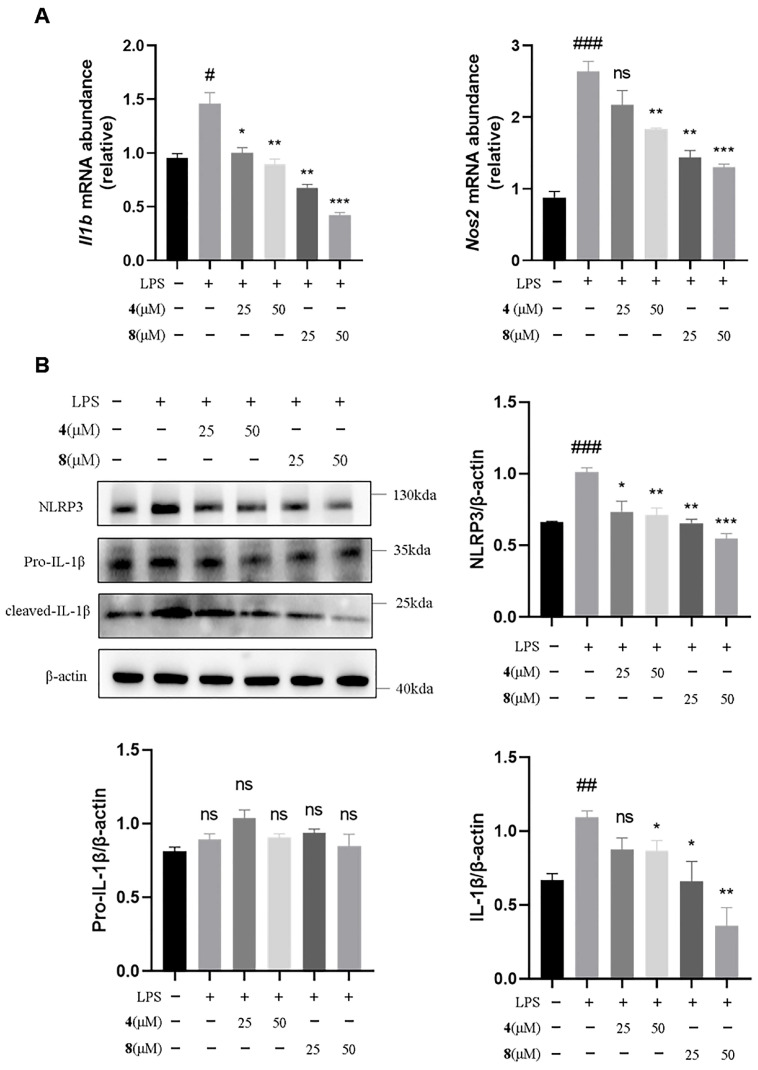
Compounds **4** and **8** inhibited the NLRP3 inflammasome activation. (**A**) qRT–PCR detection of Il-1β and Nos2 mRNA levels (*n* = 3). (**B**) Western blot detection of NLRP3, cleaved caspase 1, pro-caspase 1 and pro-IL-1β expression level in the lysates of iBMDMs (*n* = 3). Resulting data were from at least three biological replicates and are presented as mean ± standard error of the mean (SEM) (**A**,**B**); ^#^ *p* < 0.05, ^##^ *p* < 0.01, ^###^ *p* < 0.001, vs. normal group; * *p* < 0.05, ** *p* < 0.01, *** *p* < 0.001, vs. LPS group; ns: no significance. statistical analysis was performed using one-way ANOVA.

To investigate the anti-inflammatory signaling pathway of sorbicillinoid dimers, the two active trichotetronine (**4**) and trichodimerol (**8**) were selected for further investigation by an in vitro inflammatory model using LPS-induced immortalized mouse bone marrow-derived macrophages (iBMDMs). The expression levels of inflammation-related markers were assessed using quantitative real-time PCR (qRT-PCR) and Western blot analysis techniques. The results of qRT-PCR (Figure 6A) showed that, compared with the model group, Compounds **4** and **8** dose-dependently reduced the mRNA transcription levels of the inflammation-related factors interleukin-1β (Il-1β) and nitric oxide synthase 2 (Nos2). Next, we examined their effects on the NLRP3 inflammasome pathway associated with IL-1β. The results of Western blot analysis (Figure 6B) showed that both Compounds **4** and **8** dose-dependently reduced the expression of NLRP3 and inhibited the release of downstream IL-1β. Notably, Compound **8** exhibited a more pronounced reduction in the expression of these two proteins compared to Compound **4**. The combined experimental results indicate that Compounds **4** and **8** exhibit significant anti-inflammatory activity and can exert their anti-inflammatory effects by modulating NLRP3.

## 3. Materials and Methods

### 3.1. General Experimental Procedures

Optical rotations were measured in MeOH using the MCP 5100 digital polarimeter (Anton Paar, Graz, Austria). The CD spectrum was obtained on a J-810 spectropolarimeter at room temperature (Jasco, Easton, PA, USA). Nuclear magnetic resonance spectra were measured on an Avance AV500 NMR spectrometer (Bruker, Karlsruhe, Germany). High-resolution electrospray ionization mass spectrometry (HRESIMS) data was collected on a Zeno TOF 7600 mass spectrometer (AB SCIEX, Framingham, MA, USA). HPLC analyses were conducted on an Agilent 1260 system with a PDA detector (Agilent Technologies, Santa Clara, CA, USA). Preparative HPLC separation was conducted on a Waters 1525EF LC system (Waters, Milford, CT, USA). Sephadex LH-20 (25–100 μm) was purchased from GE Healthcare (GE Healthcare, Uppsala, Sweden). Silica gel (200−300 mesh and 300−400 mesh) and precoated silica gel GF254 plates were purchased from Qingdao Marine Chemical Co., Ltd. (Qingdao, China). High-glucose Dulbecco’s modified Eagle medium (DMEM) was purchased from Gibco (Shanghai, China). Fetal bovine serum (FBS) was obtained from VIVA Cell (Shanghai, China). Phosphate-buffered saline (PBS) and dimethyl sulfoxide (DMSO) were purchased from KeyGEN Bio TECH (Shanghai, China). Lipopolysaccharide (LPS) was obtained from Sigma (Shanghai, China). Nitric oxide assay kit was obtained from Beyotime (Shanghai, China). Mouse IL-6 uncoated ELISA Kit was obtained from Invitrogen (Vienna, Austria).

### 3.2. Fermentation, Extraction, Isolation, and Purification

Isolation of *Penicillium* sp. NX-S-6 and its phylogenetic characterization were described previously [20]. *Penicillium* sp. NX-S-6 was cultivated on three 250 mL Erlenmeyer flasks with 50 mL of potato dextrose broth (200 g potato infusion and 20 g dextrose in 1 L water) for 3 days at 28 °C as seed cultures. The seed cultures, each 3 mL, were moved to thirty-three 1000 mL Erlenmeyer flasks containing solid rice medium (each contained 80 g rice and 120 mL deionized water). The flasks were incubated under static conditions at room temperature for 25 days. After the fermentation, the cultures were extracted with ethyl acetate (EtOAc) three times, and the EtOAc portion was evaporated under reduced pressure to obtain a crude extract (49.7 g). The crude extract was dispersed in water and extracted with petroleum ether (PE) and EtOAc successively, to afford EtOAc extract (11.6 g). The EtOAc extract was subjected to a silica gel chromatographic column eluted with a gradient PE/EtOAc system to produce 14 fractions (Fr.1−14). Fr. 6 was recrystallized to yield Compound **13** (36.2 mg). Fr. 11 (2.5 g) was purified by a silica gel column with a gradient DCM/MeOH system to obtain subfractions Fr.11A−11J. Fr. 11C (0.6 g) was then purified by a Sephadex LH-20 column (MeOH) to obtain subfractions Fr.11C1−11C7. Fr.11C2 (325 mg) was further purified by a preparative HPLC (50% MeCN) to obtain Compounds **4** (21.7 mg), **5** (33.4 mg), **6** (24.5 mg) and **7** (40.2 mg). Fr.11C4 (93 mg) was further purified by a preparative HPLC eluted with 15~50% MeCN to obtain Compounds **8** (3.3 mg) and **14** (3.7 mg). Fr.11C5 (68 mg) was further purified by a preparative HPLC (20% MeCN) to obtain Compound **17** (3.8 mg). Fr.11E (381 mg) was purified by a Sephadex LH-20 column (MeOH) and then subjected to a preparative HPLC (35% MeCN) to obtain Compound **12** (3.9 mg). Fr.11F (396 mg) was purified by a Sephadex LH-20 column (MeOH) followed by a preparative HPLC eluted with 20% MeCN to obtain Compounds **11** (3.6 mg) and **16** (2.3 mg). Fr. 11H and 11I were combined (408 mg) and purified by a Sephadex LH-20 column (MeOH) and then subjected to a preparative HPLC (30% MeCN) to obtain Compounds **2** (4.1 mg) and **3** (2.6 mg). Fr. 12 (902 mg) was purified by Sephadex LH-20 column (100% MeOH) to obtain subfractions Fr.12A−12E. Fr.12C (540 mg) was purified by silica gel chromatographic column eluted with DCM/MeOH (200:1, 120:1 and 90:1) to obtain subfractions Fr.12C1−12C10. Fr.12C3 (45 mg) was further purified by preparative HPLC (45% MeCN) to obtain Compound **1** (1.3 mg). Fr.12C5 (130 mg) was further purified by preparative HPLC (18% MeCN) to obtain Compound **15** (2.3 mg). Fraction 13 (3.8 g) was isolated by a silica gel chromatographic column with a gradient DCM/MeOH system to obtain subfractions Fr.13A−13N. Fr.13G (322 mg) was purified by a Sephadex LH-20 column (MeOH) followed by a preparative HPLC (10% MeCN) to obtain Compound **18** (4.0 mg). Fr.13J (741 mg) was purified by a Sephadex LH-20 column (100% MeOH) to obtain subfractions Fr.13J1−13J6. Fr.13J2 (112 mg) was further purified by preparative HPLC (18% MeCN) to obtain Compound **10** (9.7 mg). Fr.13J3 (85 mg) was further purified by preparative HPLC (15% MeCN) to obtain Compound **9** (7.8 mg).

*Quinosorbicillinol (**1**)*: yellow powder; [*α*${]}_{D}^{25}$ −227.2^0^ (*c =* 0.1, MeOH); ^13^C NMR and ^1^H NMR data, see Table 1; HRESIMS *m*/*z* 405.1819 [M − H]^−^ (calcd. for C_22_H_25_N_2_O_4_, 405.1814).

*Bisorbicillpyrone B (**2**)*: yellow powder; [*α*${]}_{D}^{25}$ +163.7 (*c =* 0.2, MeOH); ^13^C NMR and ^1^H NMR data, see Table 2; HRESIMS *m*/*z* 469.1497 [M − H]^−^ (calcd. for C_25_H_25_O_9_, 469.1499).

*Citrinadin E (****10****)*: Yellow powder; [*α*${]}_{D}^{25}$ +66.5 (*c =* 0.5, MeOH); ^13^C NMR and ^1^H NMR data, see Table 2; HRESIMS *m*/*z* 466.3069 [M + Na]^+^ (calcd. for C_28_H_40_N_3_O_3_, 466.3070).

*Norcyclocitrinoic acid C (****11****)*: Colorless powder; [*α*${]}_{D}^{25}$ +188.9 (*c =* 0.1, MeOH); ^13^C NMR and ^1^H NMR data, see Table 2; HRESIMS *m*/*z* 383.1859 [M − H]^−^ (calcd. for C_23_H_27_O_5_, 383.1858), 767.3785 [2M − H]^−^ (calcd. for C_46_H_55_O_10_, 767.3795).

*Stapyrone I (****14****)*: Colorless oil; [*α*${]}_{D}^{25}$ +10.1 (*c =* 0.2, MeOH); ^13^C NMR and ^1^H NMR data, see Table 3; HRESIMS *m*/*z* 197.0818 [M − H]^−^ (calcd. for C_10_H_13_O_4_, 197.0814).

### 3.3. Quantum Chemistry Calculation

The theoretical calculations of Compounds **1**, **11** and **14** were performed using Gaussian 09 [38]. The possible conformations were initially obtained from the program Spartan’14 and then optimized at B3LYP/6-31G* level in the gas phase. Room-temperature equilibrium populations were calculated according to the Boltzmann distribution law. The ECD calculations were performed using time dependent density functional theory (TDDFT) [39] at wB97xd/6-311G** level in methanol with the PCM model. The ECD spectra of compounds were obtained by weighing the Boltzmann distribution rate of each geometric conformation, and the sigma/gamma value for processing the calculated ECD was 0.3 eV [40]. All calculated curves were shifted +20 nm to better simulate experimental spectra.

### 3.4. Anti-Inflammatory Activity

A Griess method was used to screen the isolated compounds for anti-inflammatory activity as previously reported [37,41].

Cell culture. Murine macrophage cell line RAW 264.7 and immortalized bone marrow-derived macrophages (iBMDM) were obtained from Procell Life Science and Technology (Wuhan, China). Cells were cultured with DMEM supplemented with 10% FBS. All cell culture was incubated in a humidified chamber at 37 °C with 5% CO_2_.

Cell viability assay. Cell viability was tested by the CCK-8 kit. RAW264.7 cells were seeded into a 96-well plate at a density of 5 × 10^3^ per well and subsequently maintained in a humidified atmosphere containing 5% CO_2_ at 37 °C. After 12 h, the medium was replaced with 50 μM of isolated compounds and dexamethasone (positive control) for 24 h. The 10 μL fresh CCK-8 solution was added to each well and incubated at 37 °C for 2 h. The absorbance was recorded at 450 nm wavelength using a microplate Reader (M200 Pro Nanoquant, TECAN, Männedorf, Switzerland).

Nitric oxide and IL-6 inhibitory activity. RAW 264.7 cells were cultured in 24-well flat-bottomed plates for 24 h at a density of 2 × 10^5^/mL. The medium was replaced with various concentrations (2.5, 5.0, 10, 25 and 50 μM) of isolated compounds and dexamethasone (positive control) and LPS with a final concentration of 1 μg/mL was added to stimulate RAW264.7 cells for 24 h. After incubation, the cell supernatant was collected and centrifuged at 5000 rpm for 10 min. The NO and IL-6 concentrations in the supernatant were determined using the protocol of nitric oxide assay kit (Beyotime, Shanghai, China) and Mouse IL-6 uncoated ELISA kit (Invitrogen, Vienna, Austria), respectively. The absorbance was measured by a Tecan infinite M nano plate reader (Männedorf, Switzerland) at 540 nm and 450 nm. The inhibition rate was determined by the following equation:

$$Inhibition\;rate\left(\%\right)=\frac{{OD}_{M}-{OD}_{X}}{{OD}_{M}-{OD}_{C}}\times 100\%$$

*OD_M_* stands for the absorbance of the model

*OD_X_* stands for the absorbance of the sample

*OD_C_* stands for the absorbance of the control

qRT–PCR. Total mRNA was extracted using the FreeZol kit (Vazyme Biotech Co., Ltd., Nanjing, China) according to the manufacturer’s instructions. The total mRNA was reverse-transcribed into cDNA using HiScript^®^II QRT SuperMix for qPCR (+qDNA wiper) reagent (Vazyme Biotech Co., Ltd., Nanjing, China). The real-time quantitative PCR (qRT–PCR) reaction system was prepared with ChamQ SYBR qPCR Master Mix (Without ROX) reagent (Vazyme Biotech Co., Ltd., Nanjing, China) and performed on a real-time fluorescent quantitative PCR instrument (Lighe Cycler 96, Roche, Basel, Switzerland); the mRNA expression levels were normalized using GAPDH as an internal reference [13].

Western Blot. After completing the cell modeling, the cells were collected and lysed in RIPA lysis buffer (Beyotime, Shanghai, China) containing phenylmethylsulfonyl fluoride (PMSF, Beyotime, Shanghai, China) at 4 °C for 30 min. The lysate was centrifuged at 12,000× *g* for 15 min at 4 °C. The supernatant was collected, mixed with SDS-PAGE protein loading buffer (5×) (Beyotime, Shanghai, China), and heated in a metal bath at 100 °C for 10 min to prepare the protein samples. The protein samples were separated by SDS-PAGE and transferred to PVDF membranes, which were then blotted with specific primary and secondary antibodies [13].

## 4. Conclusions

In summary, aiming to discover novel natural products from microorganisms in unique ecological environments, we selected the endophytic *Penicillium* sp. NX-S-6, which was originally isolated from a plant growing in an extreme alpine region. This strain was fermented using a 30 L solid rice medium, a widely adopted method for enhancing the production of diverse secondary metabolites due to its ability to mimic the natural habitat of endophytic fungi and promote metabolic diversity. After the fermentation process, the extracts were subjected to a series of separation techniques, including column chromatography on silica gel, Sephadex LH-20, and semi-preparative high-performance liquid chromatography (HPLC). Through these meticulous isolation steps, five new secondary metabolites were successfully obtained, including a hybrid sorbicillinoid (**1**) featuring a novel 6/6/5/6/6 fused pentacyclic carbon skeleton. This unique structure represents a significant discovery, as it expands the structural diversity of sorbicillinoids, a class of natural products known for their broad biological activities.

Bioactivity assays demonstrated that Compounds **1**, **4**, and **8** exhibited potent anti-inflammatory activity by inhibiting the release of nitric oxide (NO) and interleukin-6 (IL-6) in LPS-stimulated RAW264.7 macrophages. These results were further validated by dose–response experiments, which revealed that the inhibitory effects of these compounds were concentration-dependent. Furthermore, Compounds **4** and **8** suppressed IL-1β secretion by inhibiting the activation of the NLRP3 inflammasome, a key player in the inflammatory response. Mechanistic studies showed that these compounds regulated the phosphorylation levels of multiple signaling proteins involved in the NLRP3 pathway, indicating a multi-target mode of action.

This study not only enriches the library of natural products from endophytic fungi but also provides experimental evidence for the further development of anti-inflammatory drugs. The discovery of these bioactive compounds with novel structures highlights the potential of microorganisms from extreme environments as a valuable source for drug discovery, suggesting that exploring understudied ecological niches could lead to the identification of more promising lead compounds in the future.

## Figures and Tables

**Table 1:** ^1^H (500 MHz) and ^13^C (125 MHz) NMR data for quinosorbicillinol (**1**) in CDCl_3_.

No.	*δ*_C_, Type	*δ*_H_ (*J* in Hz)	No.	*δ*_C_, Type	*δ*_H_ (*J* in Hz)
2	162.3, C		4′	26.9, CH_2_	2.41, m
3	105.3, C		5′	39.4, CH_2_	2.17, t (7.8)
4	155.9, C		6′	89.2, C	
4a	114.9, C		7′	31.1, CH_2_	2.08, m 2.62, dd (14.0, 2.3)
5	122.7, CH	7.84, d (7.9)	8′	34.7, CH	3.84, brs
6	121.8, CH	7.23, t (7.6)	9′	82.0, C	
7	131.3, CH	7.59, t (7.7)	10′	165.6, C	
8	114.5, CH	7.34, d (8.5)	11′	101.2, C	
8a	139.6, C		12′	173.5, C	
9	29.9, CH_3_	3.70, s	13′	26.0, CH_3_	1.75, s
1′	18.1, CH_3_	1.73, d (6.2)	14′	6.5, CH_3_	1.51, s
2′	127.0, CH	5.65, m	N-H		4.94, brs
3′	129.9, CH	5.58, m			

**Table 2:** ^1^H (500 MHz) and ^13^C (125 MHz) NMR data for Compounds **2**, **10** and **11**.

No.	2 ^a^		10 ^b^		11 ^a^	
	*δ*_C_, type	*δ*_H_ (*J* in Hz)	*δ*_C_, type	*δ*_H_ (*J* in Hz)	*δ*_C_, type	*δ*_H_ (*J* in Hz)
1	64.3, C			9.80, s	149.8, CH	6.09, d (13.0)
2	199.1, C		183.5, C		130.6, CH	5.78 ^c^
3	109.1, C		60.2, C		204.0, C	
3a			132.4, C			
4	46.2, CH	3.67, d (2.7)	133.0, CH	7.85, d (7.6)	47.7, CH_2_	2.67, d (17.9) 3.43, d (17.9)
5	74.9, C		122.4, CH	7.16, t (7.8)	44.0, CH	2.93, m
6	210.4, C		128.0, CH	7.72, d (8.0)	204.8, C	
7	48.8, CH	3.21, dd (10.1, 5.5)	117.2, C		125.4, CH	5.76 ^c^
7a			143.2, C			
8	45.7, CH	3.46, dd (5.5, 2.7)	41.0, CH_2_	2.20 d (15.0) 2.59 d (15.0)	157.3, C	
9	170.0, C		64.2, C		59.1, CH	2.57, m
10	119.5, CH	6.42 ^c^	57.5, CH_2_	3.24, d (12.7) 3.62, d (12.7)	74.9, C	
11	143.6, CH	7.34, dd (14.9, 15.0)			25.6, CH_2_	1.84, m 2.37, d (13.7)
12	140.5, CH	6.25 ^c^	58.6, CH	3.78, m	39.5, CH_2_	1.66 ^c^, 2.02 ^c^
13	132.3, CH	6.42 ^c^	29.5, CH_2_	1.73, 2.34, m	50.9, C	
14	18.9, CH_3_	1.92, d (7.5)	17.1, CH_2_	1.72, m	58.7, CH	2.49, dd (11.6, 6.7)
15	176.1, C		30.5, CH_2_	1.92, m	23.6, CH_2_	1.66 ^c^, 1.78, m
16	135.9, CH	6.27 ^c^	58.9, CH	3.29, m	25.5, CH_2_	1.90, m, 2.01 ^c^
17	126.1, CH	6.18, d (15.3)	25.9, CH_2_	1.77, 2.13, m	61.7, CH	2.60, m
18	156.7, C		53.7, CH	2.83, dd (13.6, 2.8)	35.1, CH_2_	2.07, d (15.4) 3.11, dd (15.4, 7.9)
19	102.6, CH	6.09, s	47.5, C		13.9, CH_3_	0.66, s
20	167.2, C		195.0, C		160.2, C	
21	101.3, C		64.5, CH	4.05, s	20.7, CH_3_	2.22, s
22	167.7, C		61.8, C		118.0, CH	5.78 ^c^
23	11.0, CH_3_	1.11, s	18.7, CH_3_	1.22, s	170.2 C	
24	23.7, CH_3_	1.25, s	24.4, CH_3_	1.61, s		
25	8.6, CH_3_	1.91, s				
26			29.6, CH_3_	2.65, s		
27			11.8, CH_3_	1.43, d (6.8)		
28			23.5, CH_3_	1.14, s		
29			24.7, CH_3_	0.93, s		

^a^ measured in CD_3_OD; ^b^ measured in CDCl_3_; ^c^ overlapped signals.

**Table 3:** ^1^ H (500 MHz) and ^13^C (125 MHz) NMR data for stapyrone I (**14**) in CDCl_3_.

No.	*δ*_C_, Type	*δ*_H_ (*J* in Hz)	No.	*δ*_C_, Type	*δ*_H_ (*J* in Hz)
2	72.7, CH_2_	3.97, t (10.9) 4.36, dd (10.9, 5.2)	7	27.1, CH_2_	2.70, m
3	38.9, CH	2.55, m	8	30.2, CH_2_	2.65, m
4	195.4, C		9	177.2, C	
5	110.0, C		10	11.6, CH_3_	1.10, d (7.1)
6	169.4, C		11	9.4, CH_3_	1.75, s

**Table 4:** Anti-inflammatory effects (IC_50_ ± SD) of active compounds.

Compounds	NO	IL-6
**1**	34.28 ± 4.00	13.51 ± 6.70
**2**	56.71 ± 0.22	≥100
**4**	17.11 ± 9.28	28.36 ± 4.79
**6**	70.24 ± 18.73	≥100
**8**	6.63 ± 1.13	13.59 ± 0.79
**9**	36.84 ± 2.55	≥100
**11**	53.86 ± 12.55	≥100
Dexamethasone	5.24 ± 0.68	7.80 ± 0.92

## Data Availability

The authors declare that all relevant data supporting the results of this study are available within the article and its Supplementary Materials file, or from the corresponding authors upon request.

## Supplementary Materials

The following are available online at https://www.mdpi.com/article/10.3390/md23070280/s1, Figures S1–S35: 1D, 2D NMR, and HRESIMS spectra of compounds **1**, **2**, **10**, **11** and **14**, Tables S1–S7: Conformational analysis of the optimized isomers and the coordinates of the optimized conformers of compounds **1**, **11** and **14**, Figure S36: Uncropped images of gel/blot. Scheme S1: Proposed biosynthetic pathway of quinosorbicillinol (**1**).
