# Supplementary material for: Anti-Inflammatory Secondary Metabolites from Penicillium sp. NX-S-6
Source: Mar Drugs. 2025 Jul 4;23(7):280. doi: 10.3390/md23070280 (PMC12300202; doi:10.3390/md23070280)
Supplement: Supplementary file 1 [file marinedrugs-23-00280-s001.zip › marinedrugs-3727537-supplementary.pdf]

Supporting Information

Anti-inflammatory secondary metabolites from *Penicillium* sp. NX-S-6

Hanyang Peng [1,†], Jiawen Sun [1,†], Rui Zhang [1], Yuxuan Qiu [1], Yu Hong [1], Fengjuan Zhou [1], Chang Wang [1], Yang Hu [1,*] and Xiachang Wang [1,2,*]

[1]*Jiangsu Key Laboratory for Functional Substances of Chinese Medicine, Nanjing University of Chinese Medicine, Nanjing 210023, China*
[2]*Fujian Province Key Laboratory for the Development of Bioactive Material from Marine Algae, College of Oceanology and Food Science, Quanzhou Normal University, Quanzhou 362000, China*
**Correspondence: xiachangwang@njucm.edu.cn (X.W.); huyang@njucm.edu.cn (Y.H.)*
†These authors contributed equally to this work

**Contents**	**Page**
**Figures S1-S35.** NMR and MS spectra of isolated compounds.	S2-S19
**Table S1**. Important thermodynamic parameters and Boltzmann distributions of the optimized **1a**, **1b**, **11a**, **11b**, **14a** and **14b** at b3lyp/6-31g* level in gas phase.	S19
**Table S2**. The coordinates for the lowest energy conformers of **1a**.	S20
**Table S3**. The coordinates for the lowest energy conformers of **1b**.	S22
**Table S4**. The coordinates for the lowest energy conformers of **11a**.	S24
**Table S5**. The coordinates for the lowest energy conformers of **11b**.	S27
**Table S6**. The coordinates for the lowest energy conformers of **14a**.	S29
**Table S7**. The coordinates for the lowest energy conformers of **14b**.	S30
**Figure S36**. Uncropped images of gel/blot.	S32
**Scheme S1.** Proposed biosynthetic pathway of quinosorbicillinol (**1**).	S33

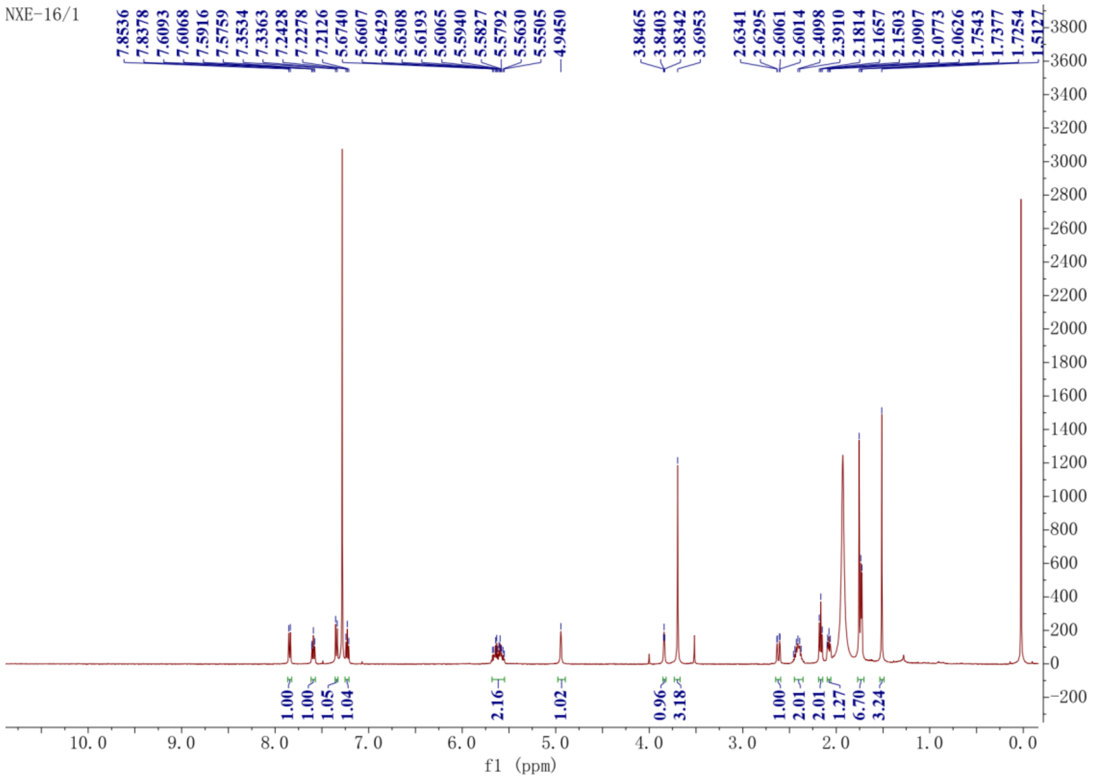

Figure S1. ^{1}H NMR spectrum (500 MHz, $CDCl_3$) of compound **1**

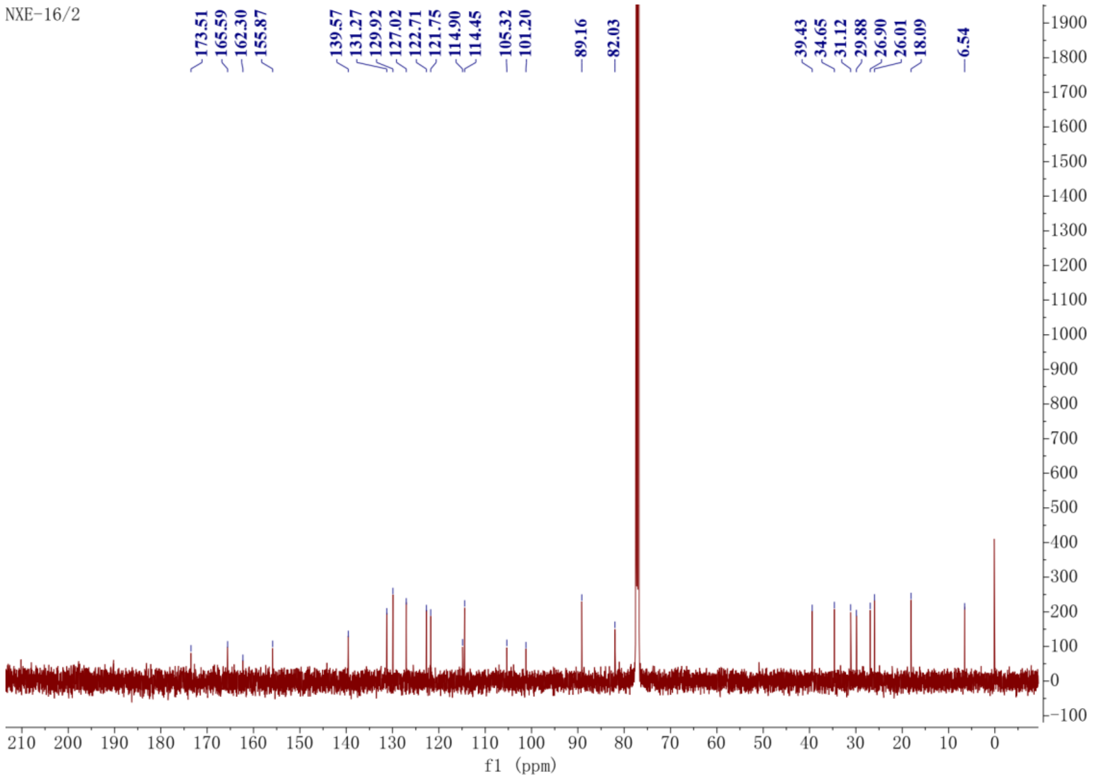

Figure S2. ^{13}C NMR spectrum (125 MHz, $CDCl_3$) of compound **1**

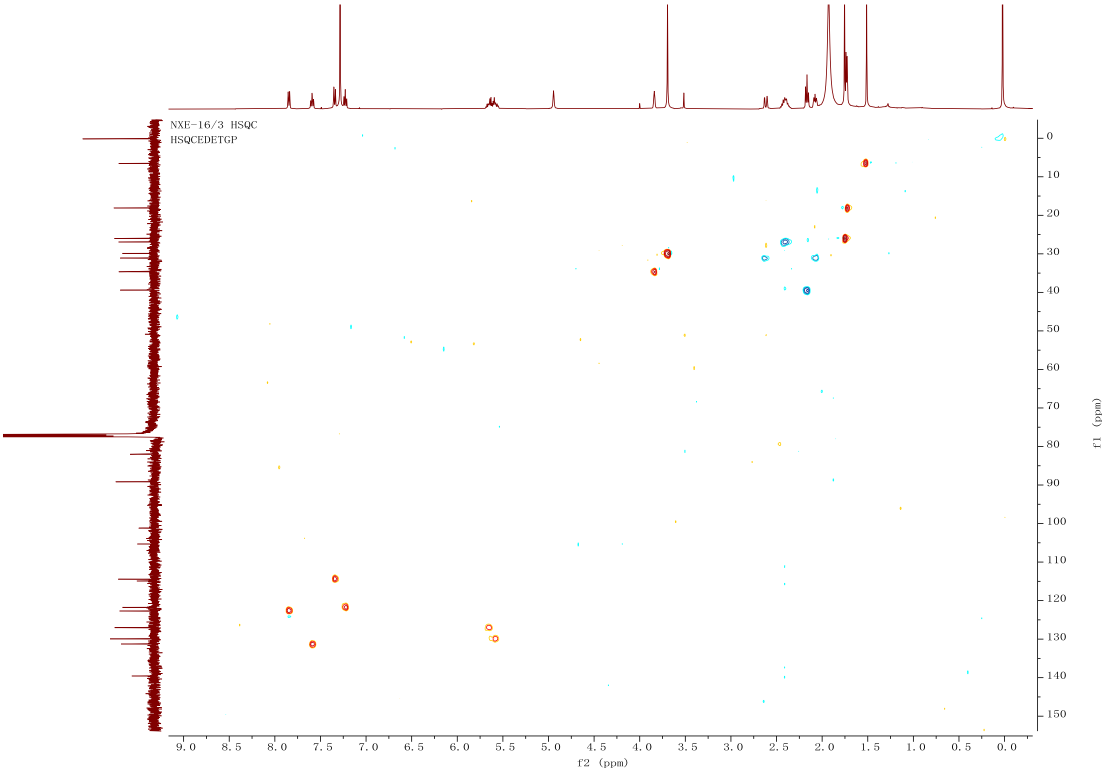

Figure S3. HSQC spectrum (500 MHz, $CDCl_3$) of compound **1**

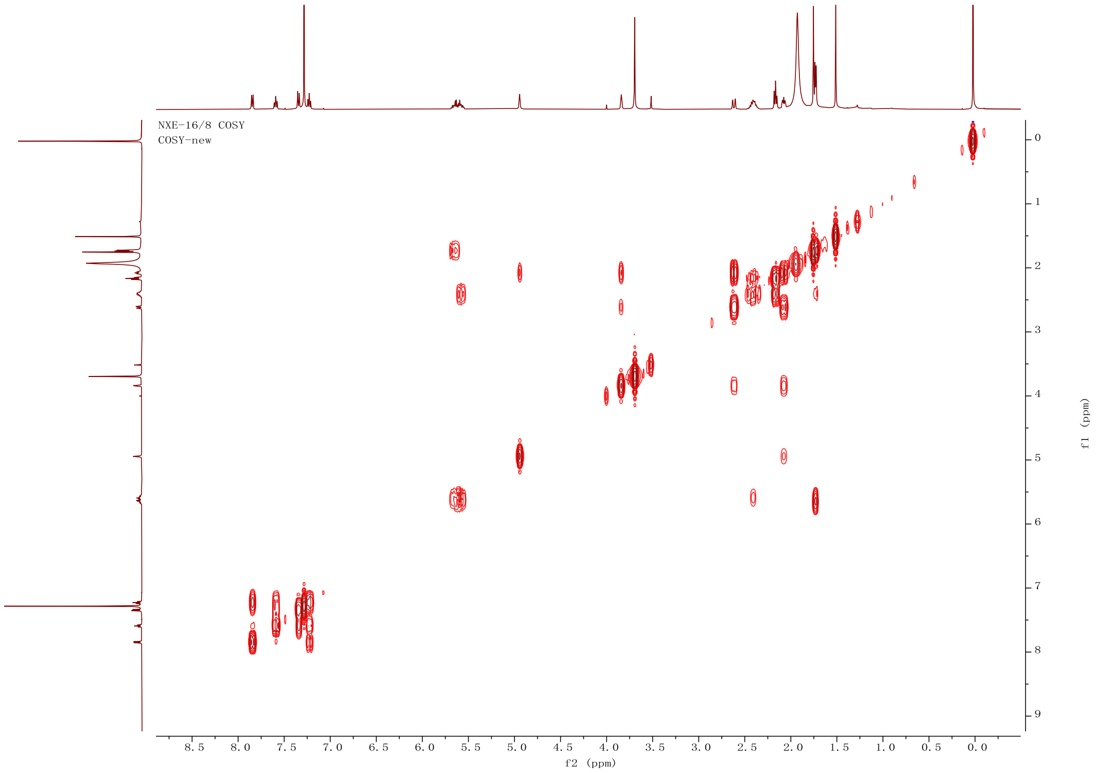

Figure S4. ^1H-^1H COSY spectrum (500 MHz, $CDCl_3$) of compound **1**

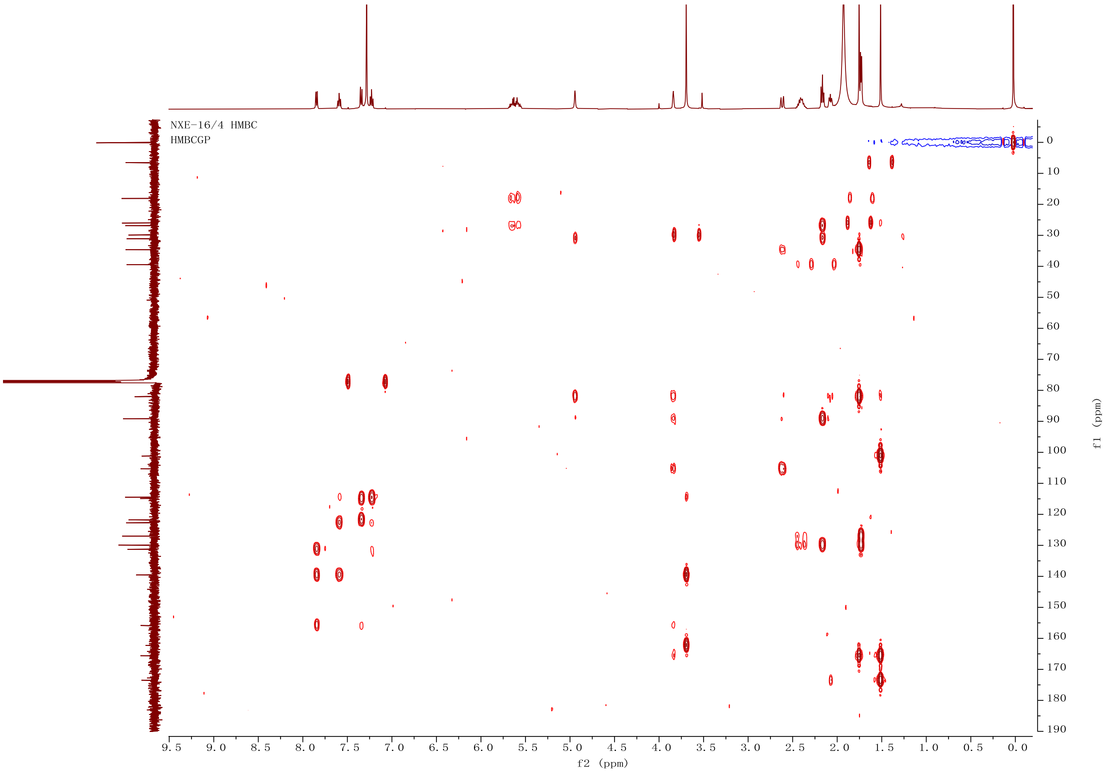

Figure S5. HMBC spectrum (500 MHz, $CDCl_3$) of compound **1**

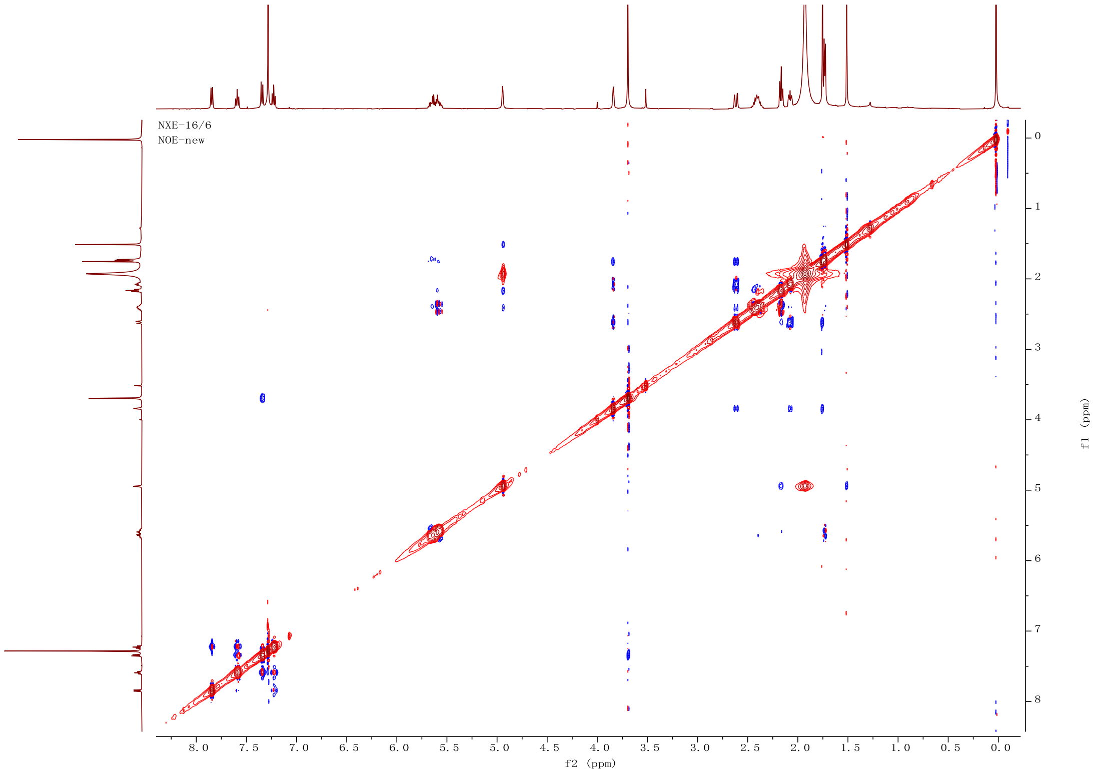

Figure S6. ROESY spectrum (500 MHz, $CDCl_3$) of compound **1**

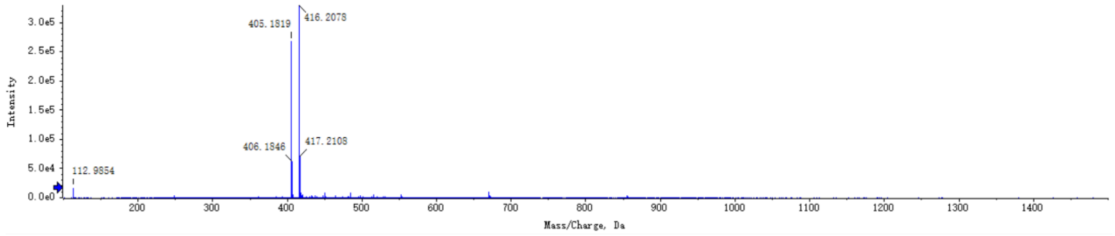

Figure S7. (−)-HRESIMS spectrum of compound **1**

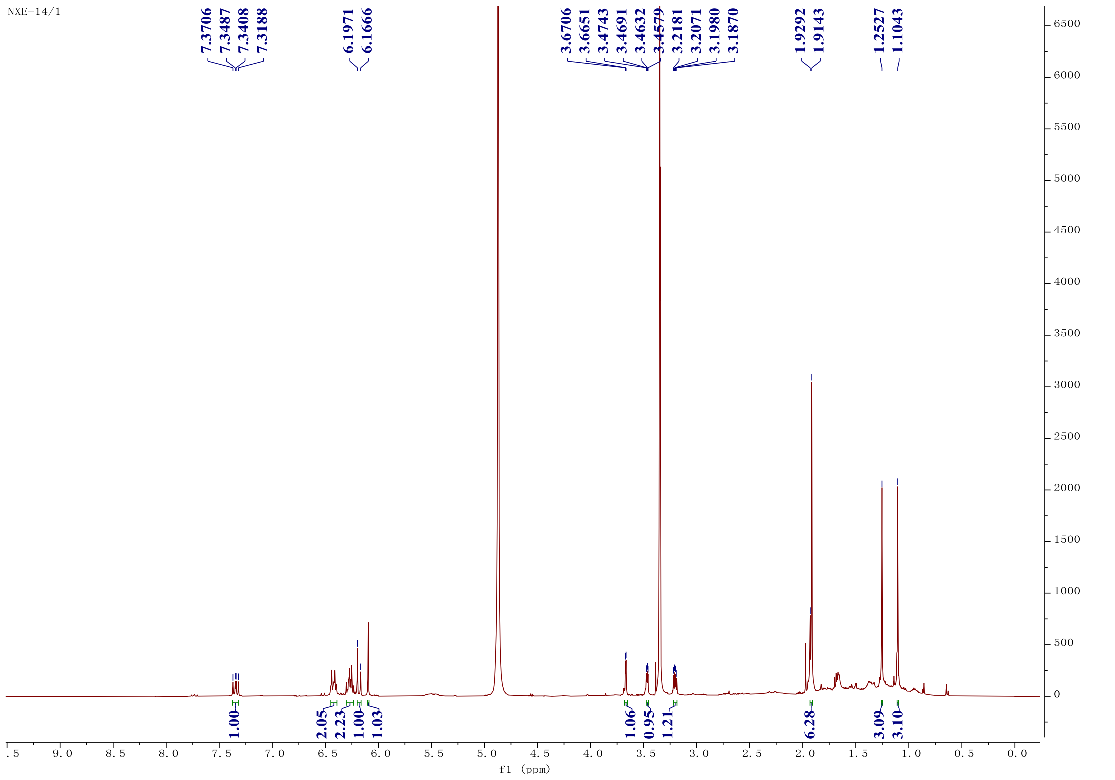

Figure S8. ^{1}H NMR spectrum (500 MHz, CD_3OD) of compound **2**

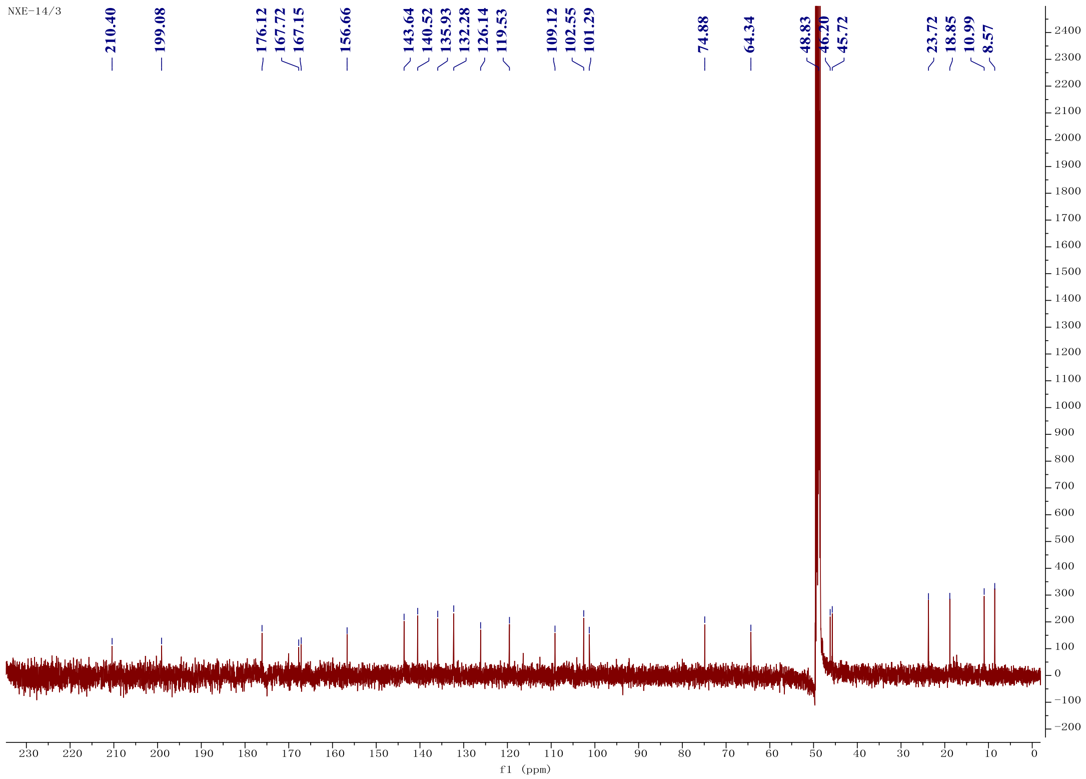

Figure S9. ^{13}C NMR spectrum (125 MHz, CD_3OD) of compound **2**

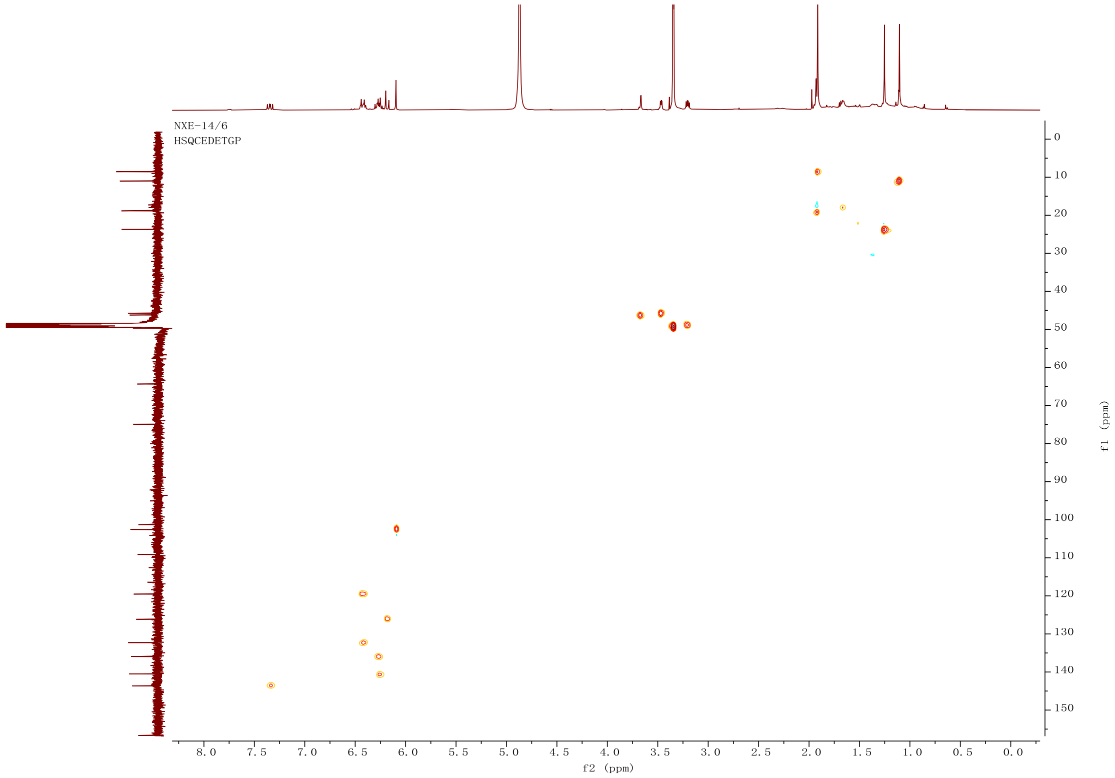

Figure S10. HSQC spectrum (500 MHz, CD_3OD) of compound **2**

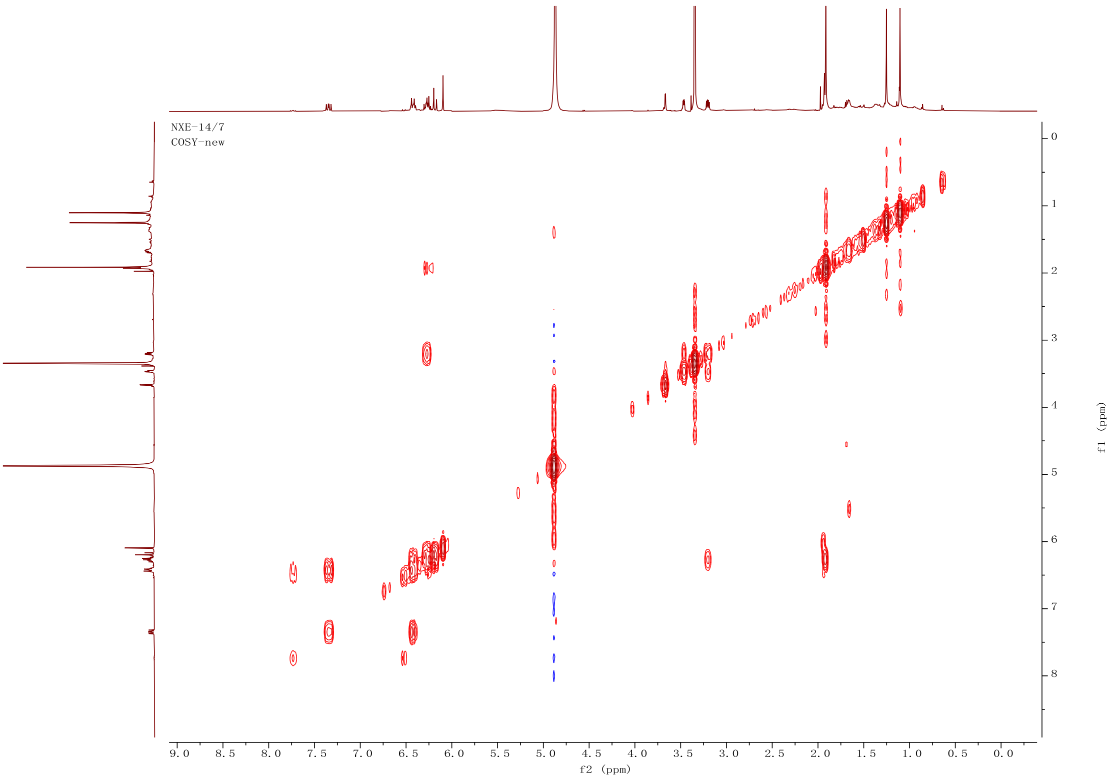

Figure S11. ^{1}H-^{1}H COSY spectrum (500 MHz, CD_3OD) of compound **2**

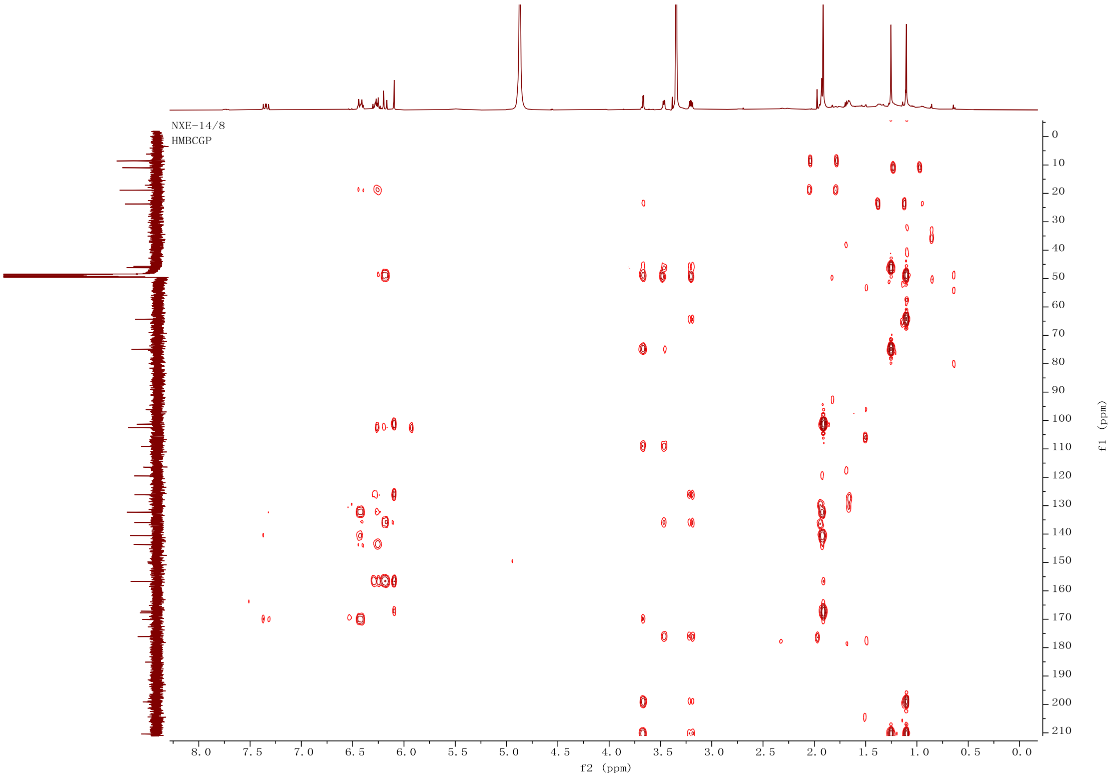

Figure S12. HMBC spectrum (500 MHz, CD_3OD) of compound **2**

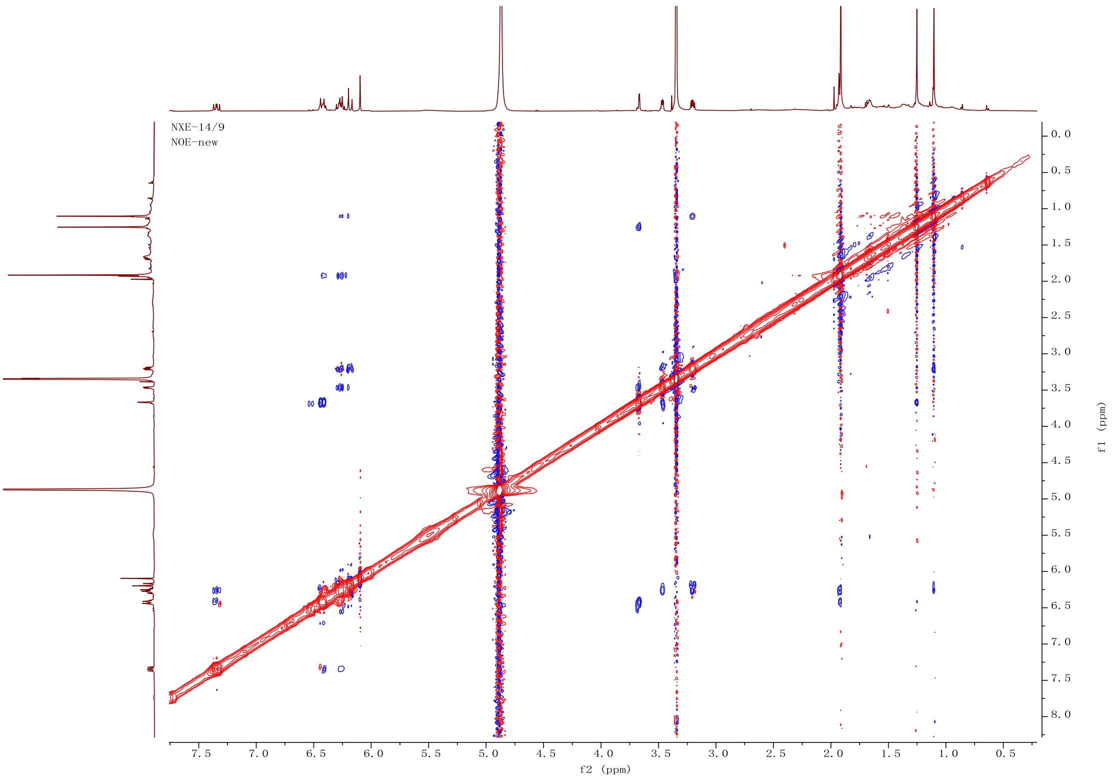

Figure S13. ROESY spectrum (500 MHz, CD_3OD) of compound **2**

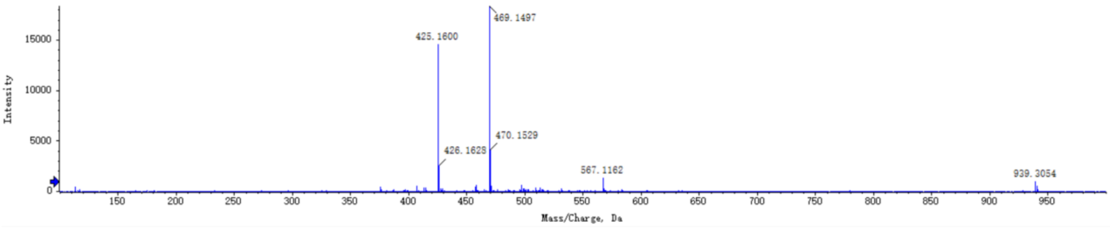

Figure S14. (−)-HRESIMS spectrum of compound **2**

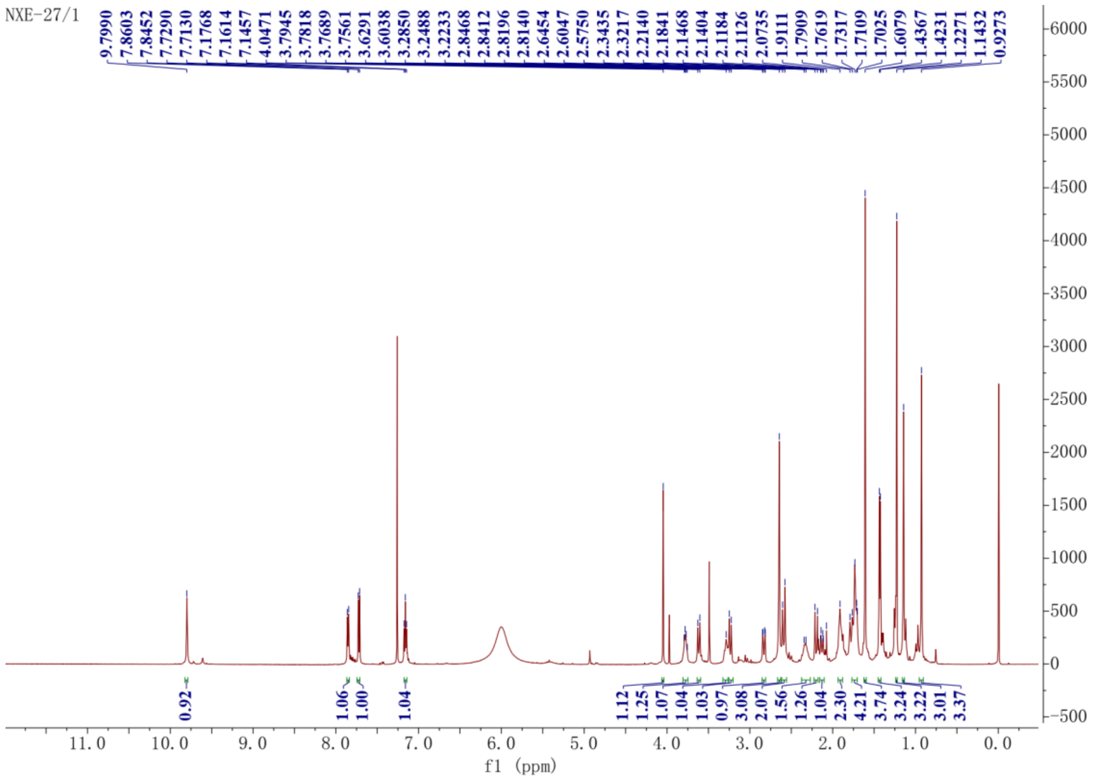

Figure S15. ^{1}H NMR spectrum (500 MHz, $CDCl_3$) of compound **10**

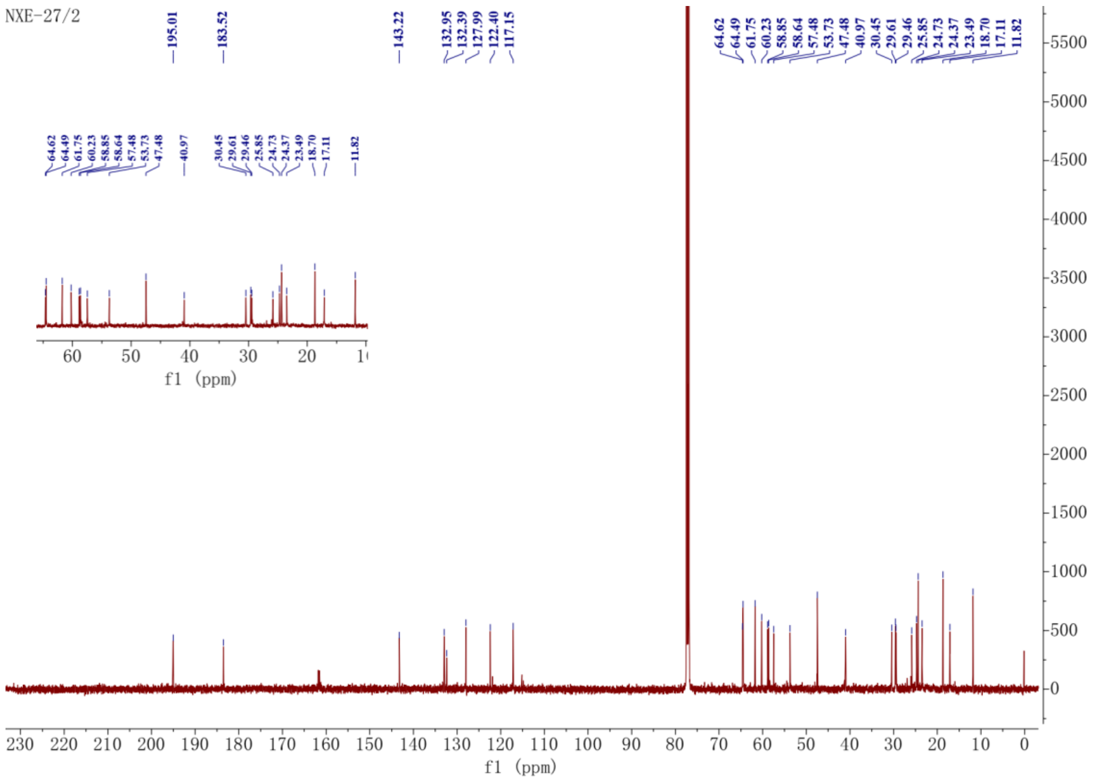

Figure S16. ^{13}C NMR spectrum (125 MHz, $CDCl_3$) of compound **10**

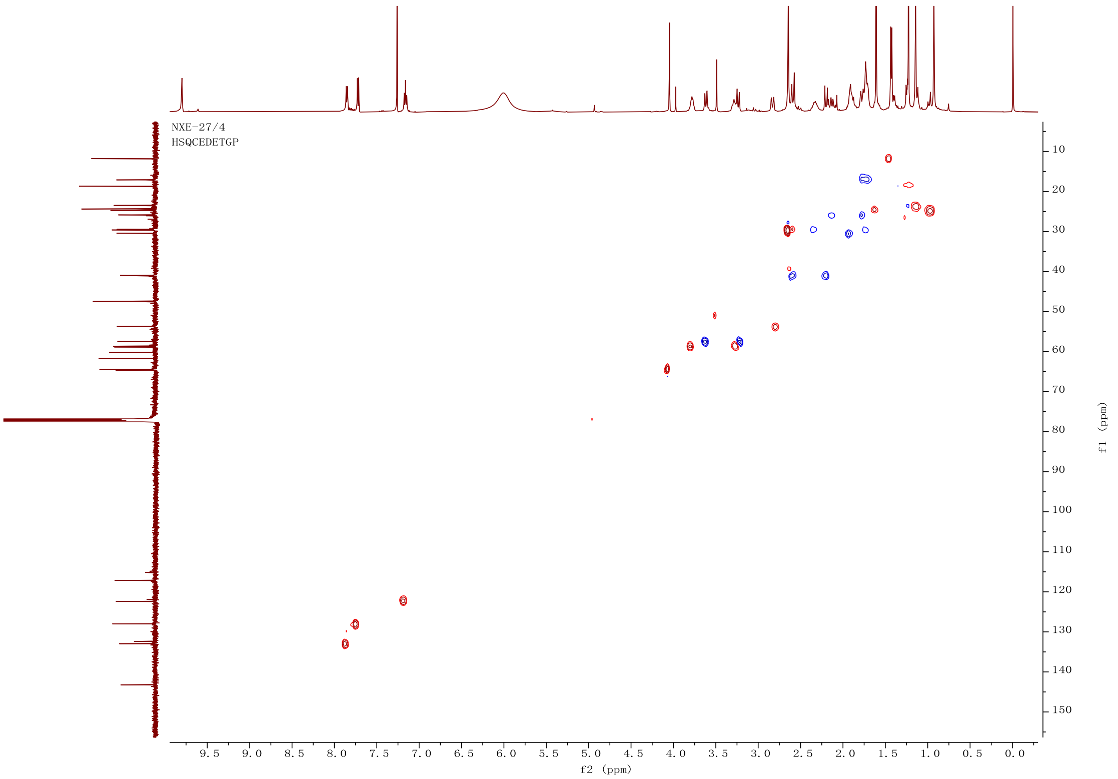

Figure S17. HSQC spectrum (500 MHz, $CDCl_3$) of compound **10**

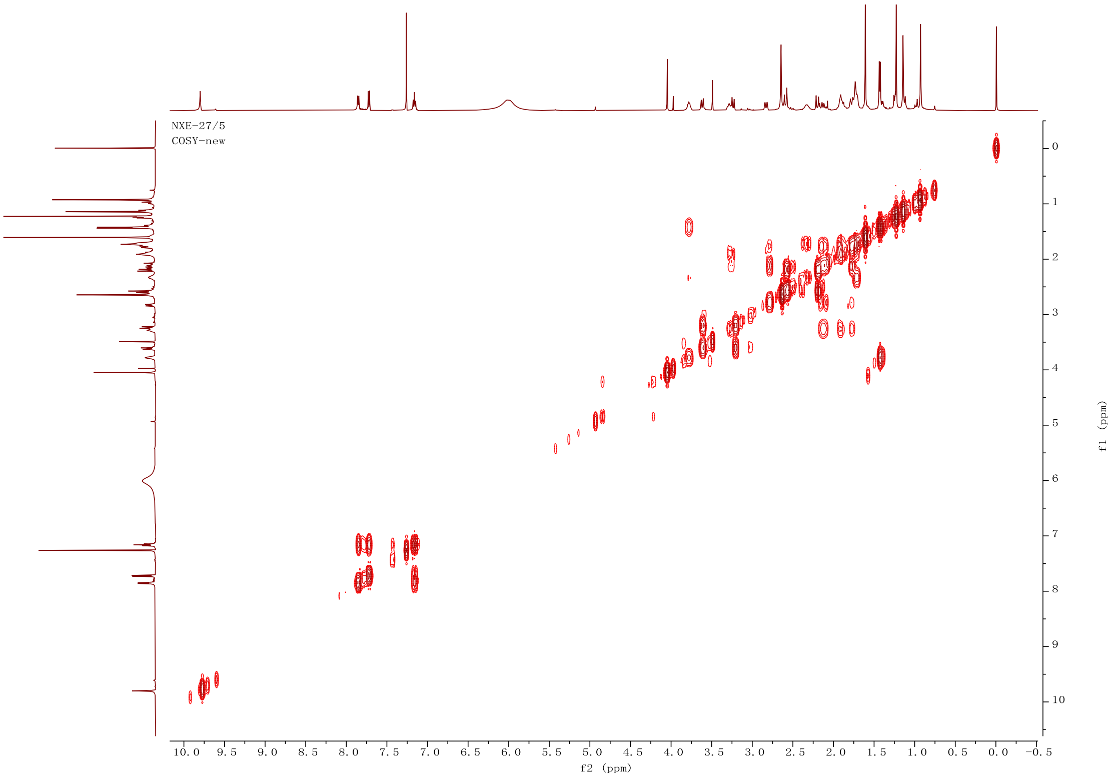

Figure S18. ^1H-^1H COSY spectrum (500 MHz, $CDCl_3$) of compound **10**

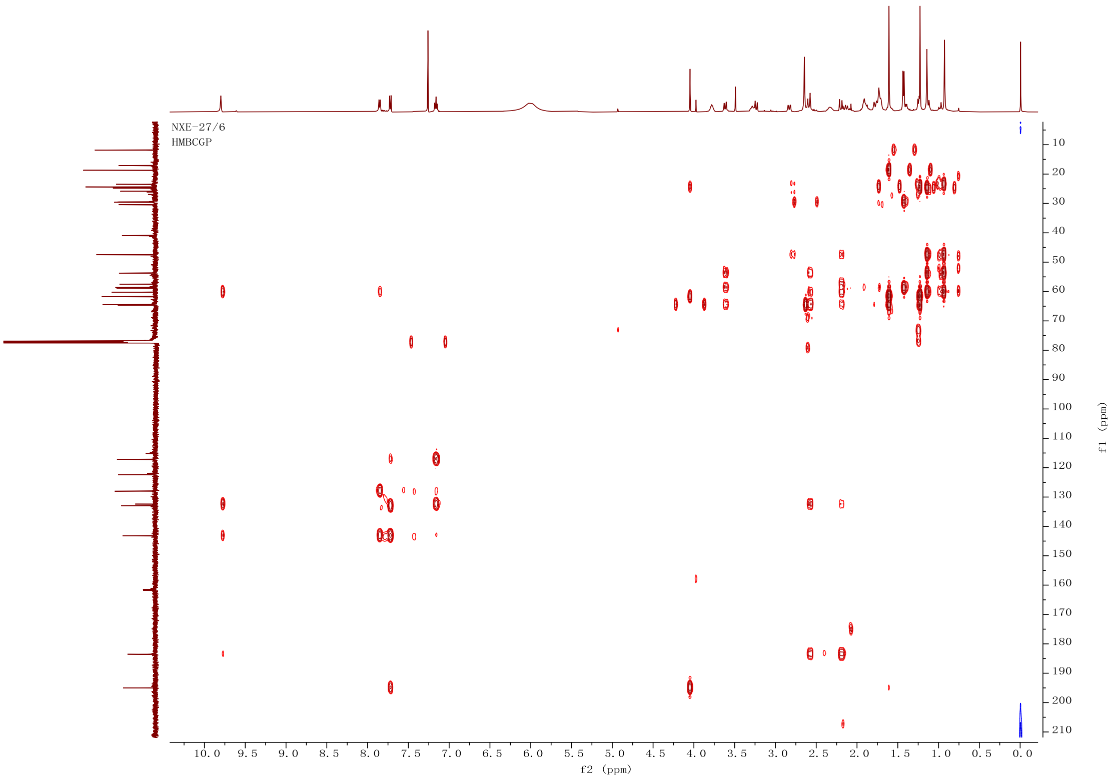

Figure S19. HMBC spectrum (500 MHz, $CDCl_3$) of compound **10**

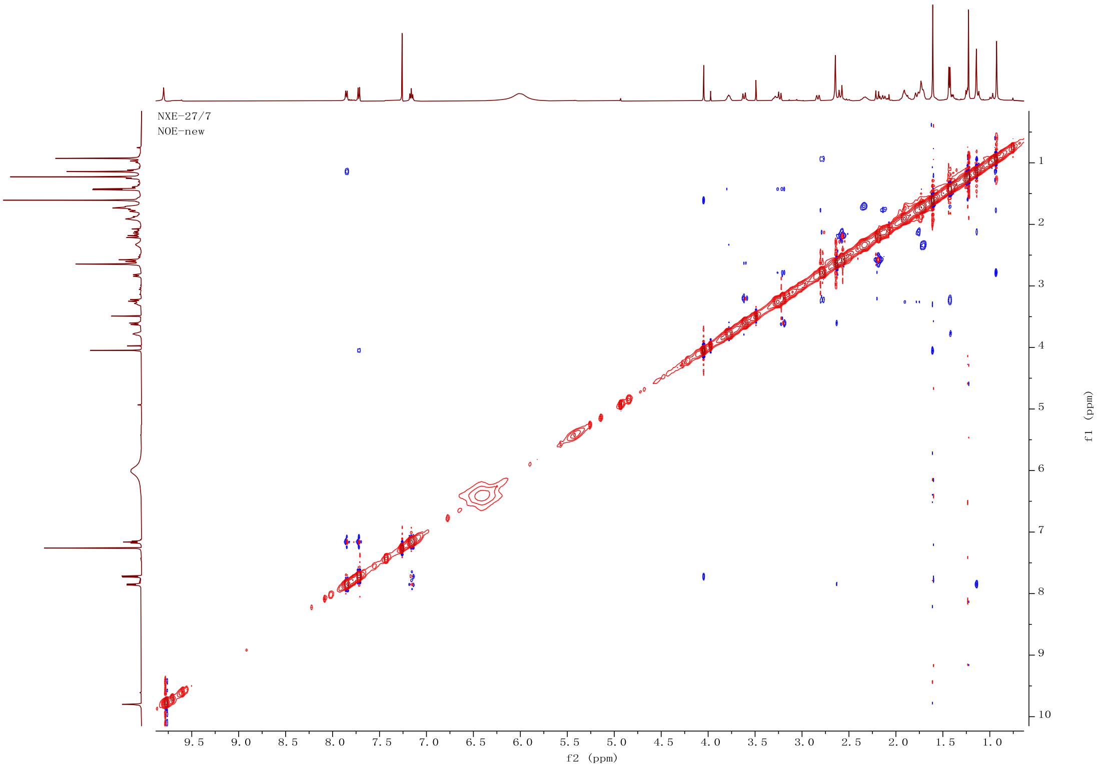

Figure S20. ROESY spectrum (500 MHz, $CDCl_3$) of compound **10**

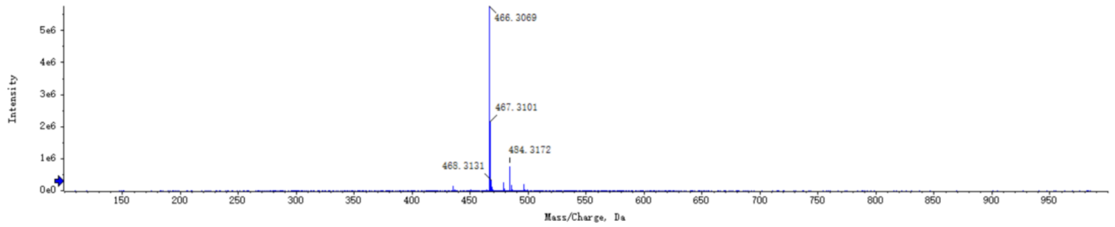

Figure S21. (+)-HRESIMS spectrum of compound **10**

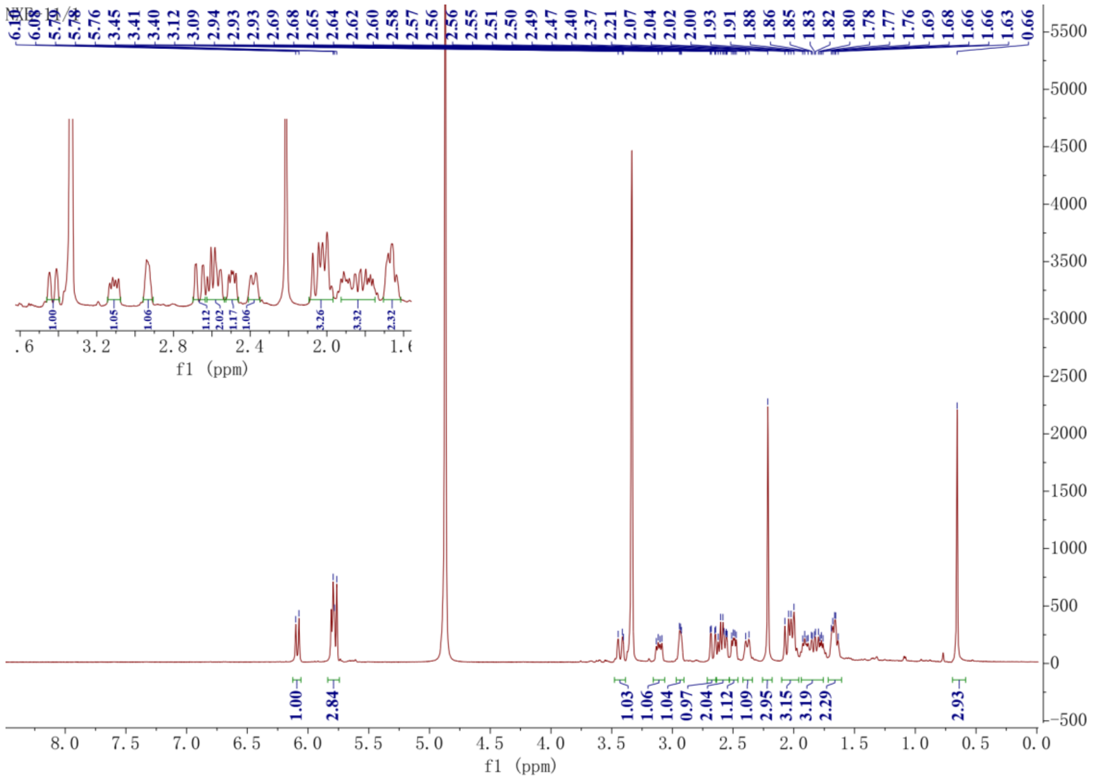

Figure S22. ^{1}H NMR spectrum (500 MHz, CD_3OD) of compound **11**

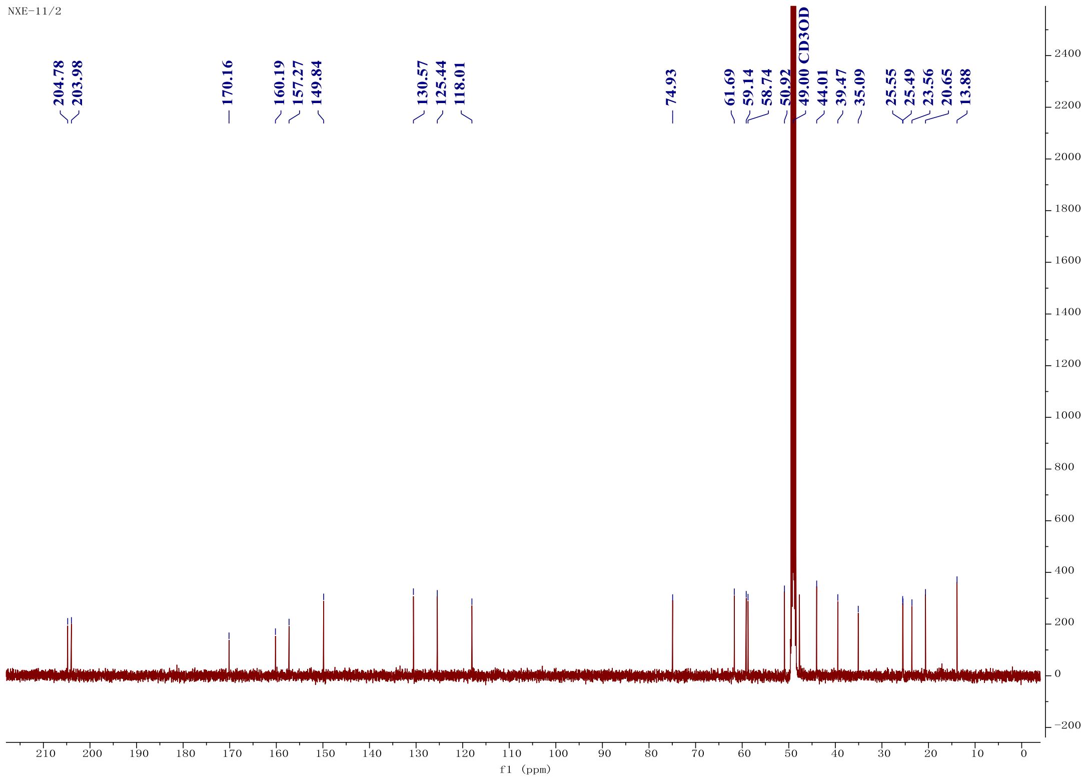

Figure S23. ^{13}C NMR spectrum (125 MHz, CD_3OD) of compound **11**

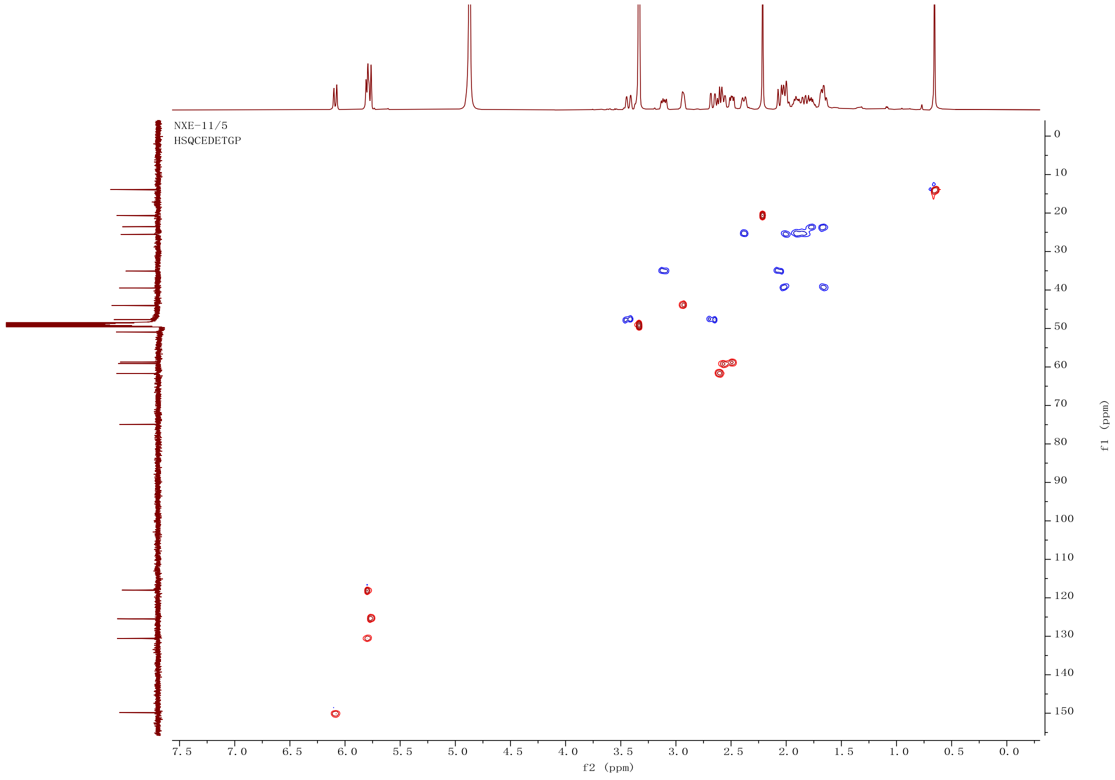

Figure S24. HSQC spectrum (500 MHz, CD_3OD) of compound **11**

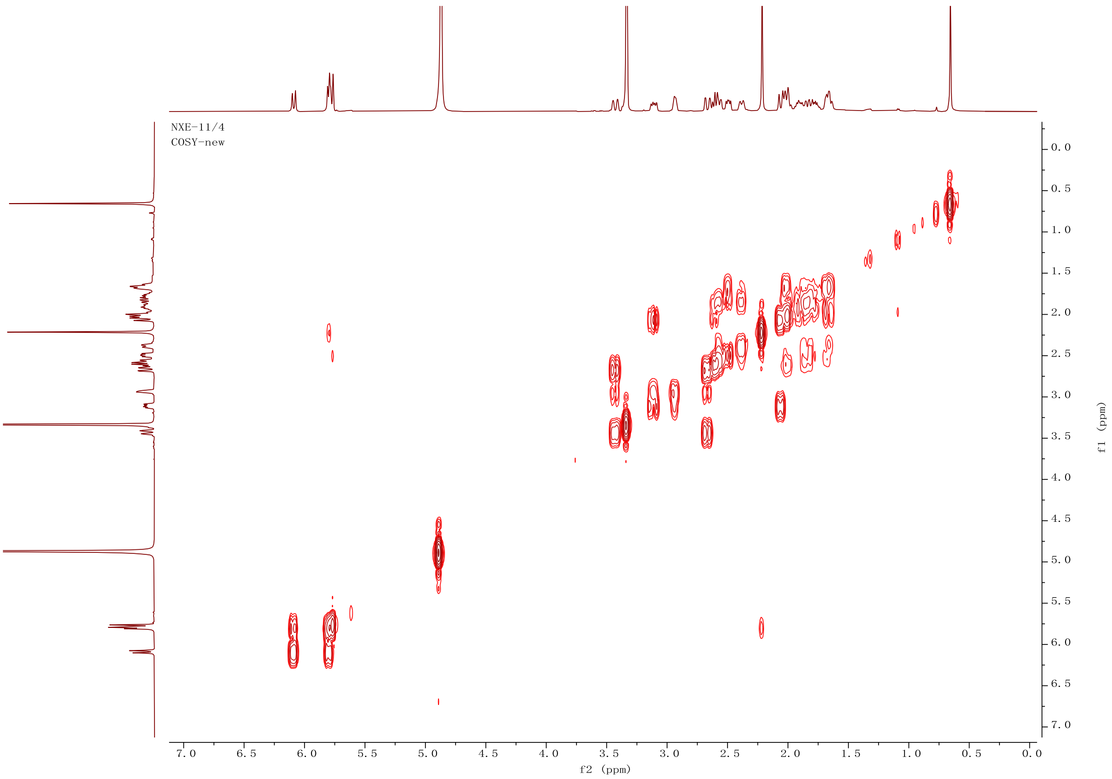

Figure S25. ^{1}H-^{1}H COSY spectrum (500 MHz, CD_3OD) of compound **11**

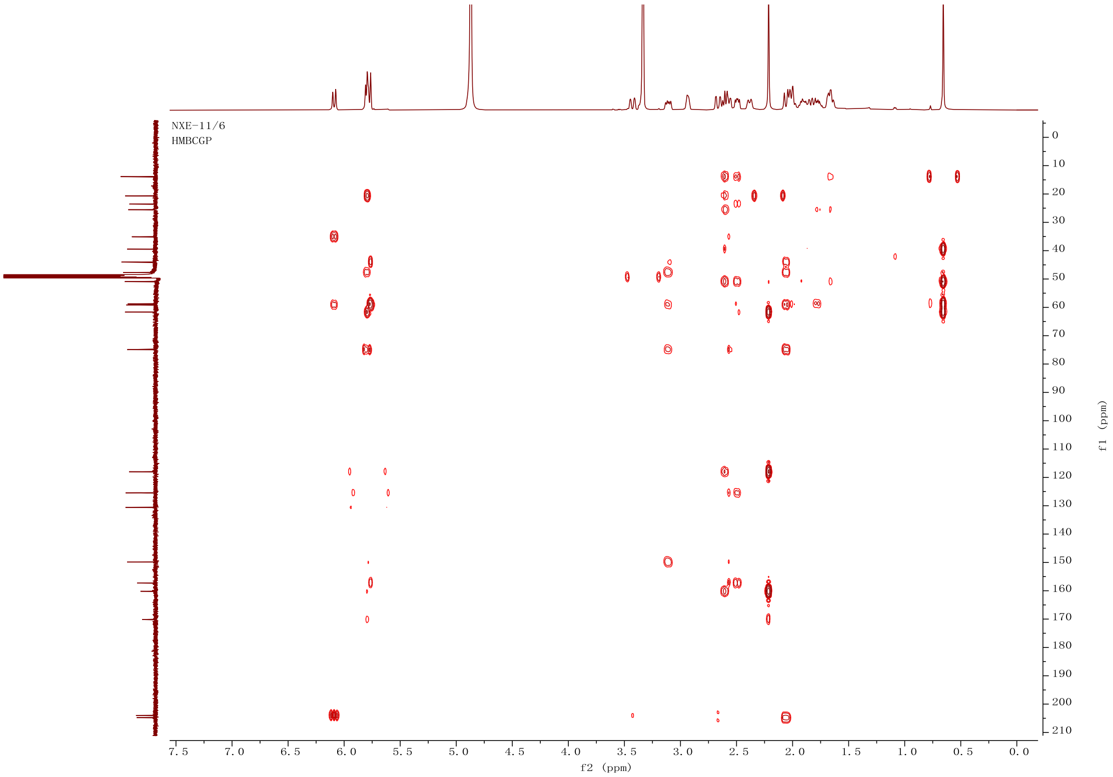

Figure S26. HMBC spectrum (500 MHz, CD_3OD) of compound **11**

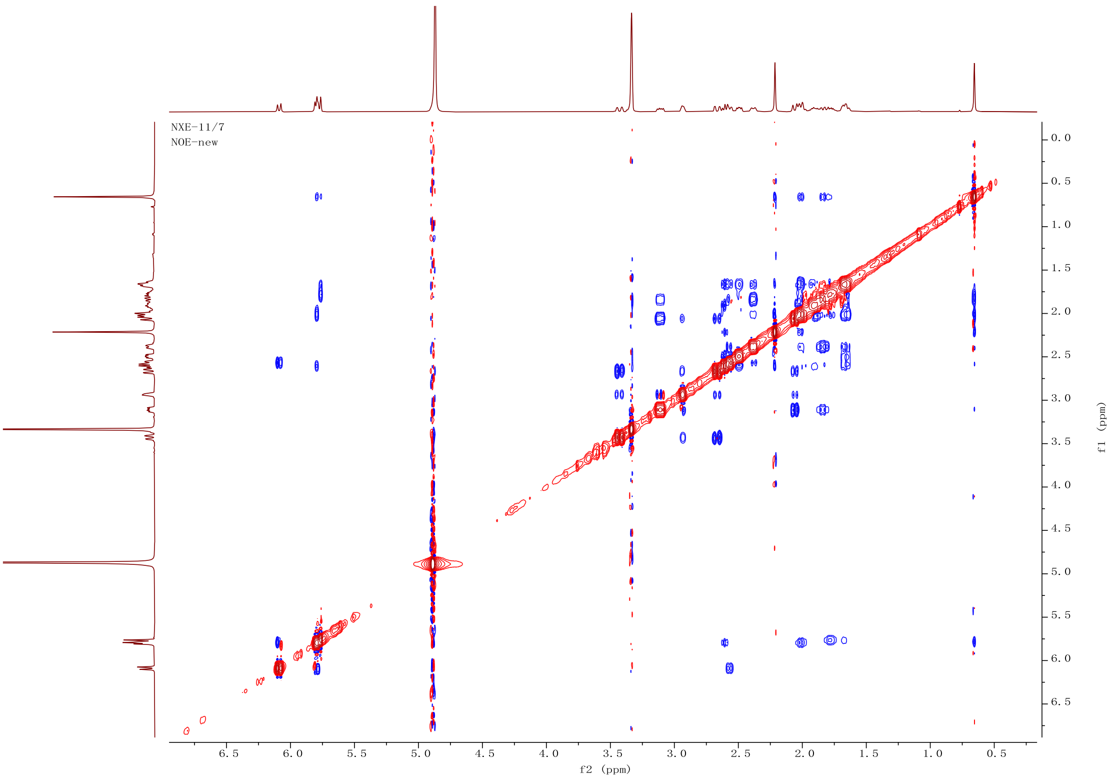

Figure S27. ROESY spectrum (500 MHz, CD_3OD) of compound **11**

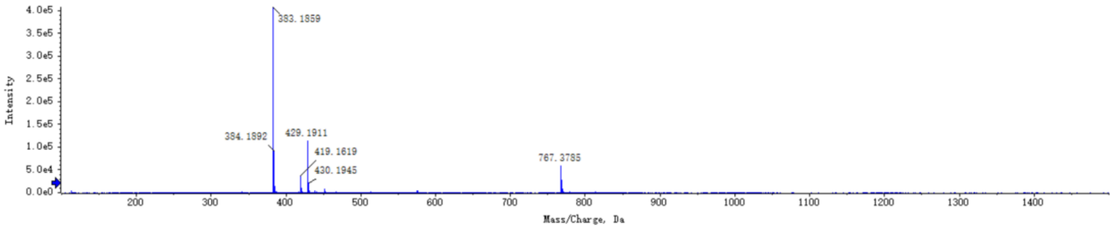

Figure S28. (−)-HRESIMS spectrum of compound **11**

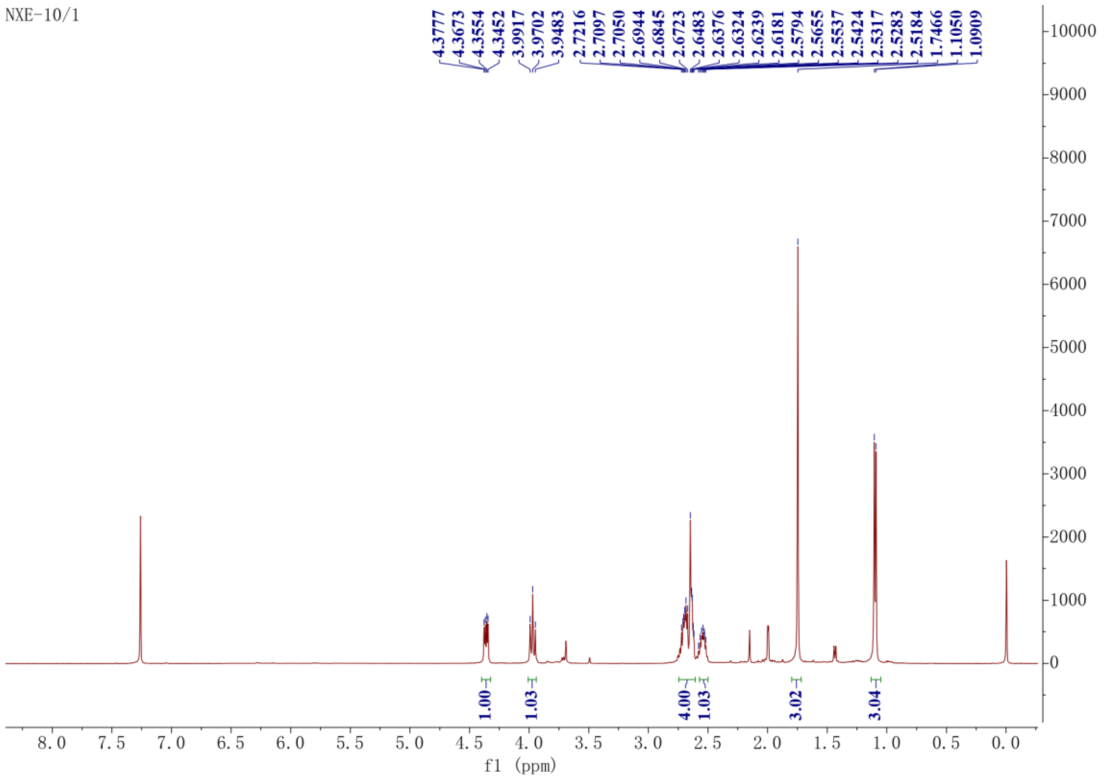

Figure S29. ^{1}H NMR spectrum (500 MHz, $CDCl_3$) of compound **14**

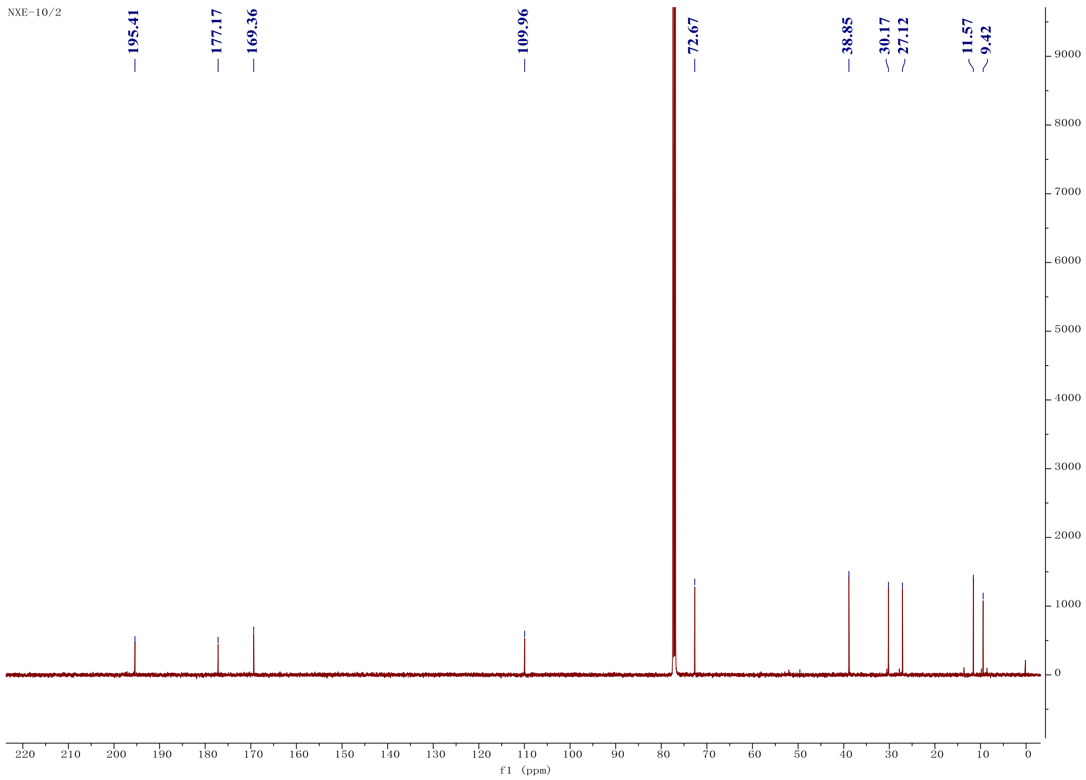

Figure S30. ^{13}C NMR spectrum (125 MHz, $CDCl_3$) of compound **14**

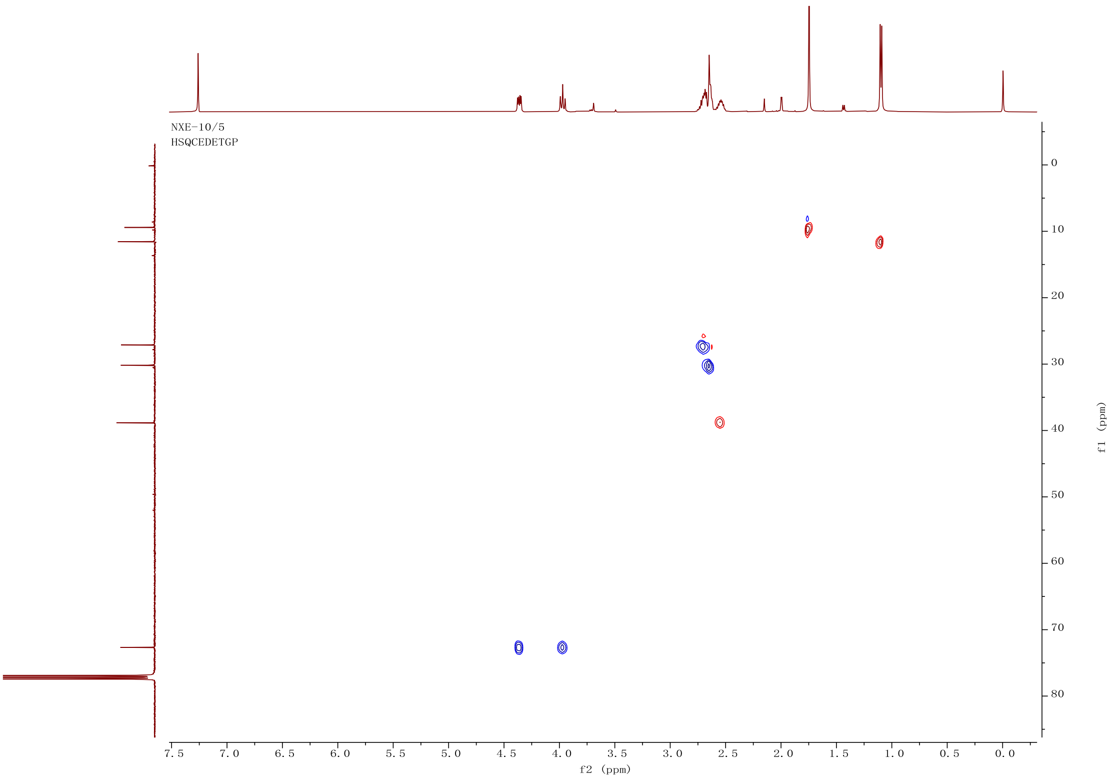

Figure S31. HSQC spectrum (500 MHz, $CDCl_3$) of compound **14**

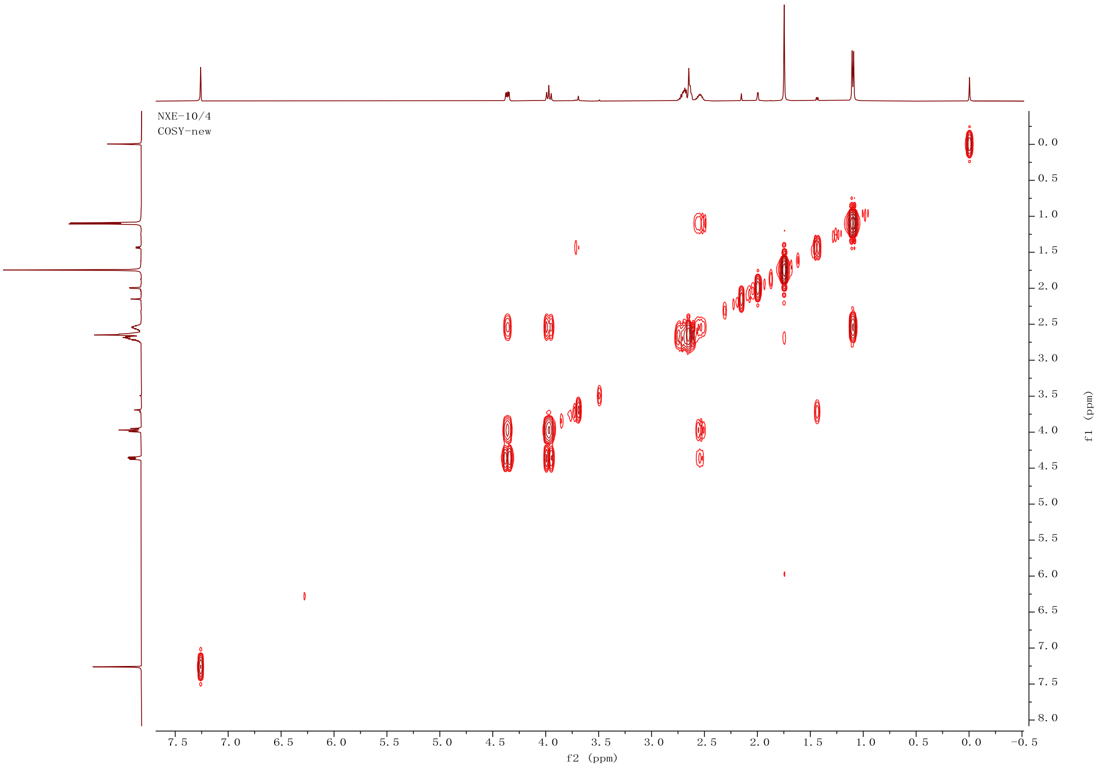

Figure S32. ^1H-^1H COSY spectrum (500 MHz, $CDCl_3$) of compound **14**

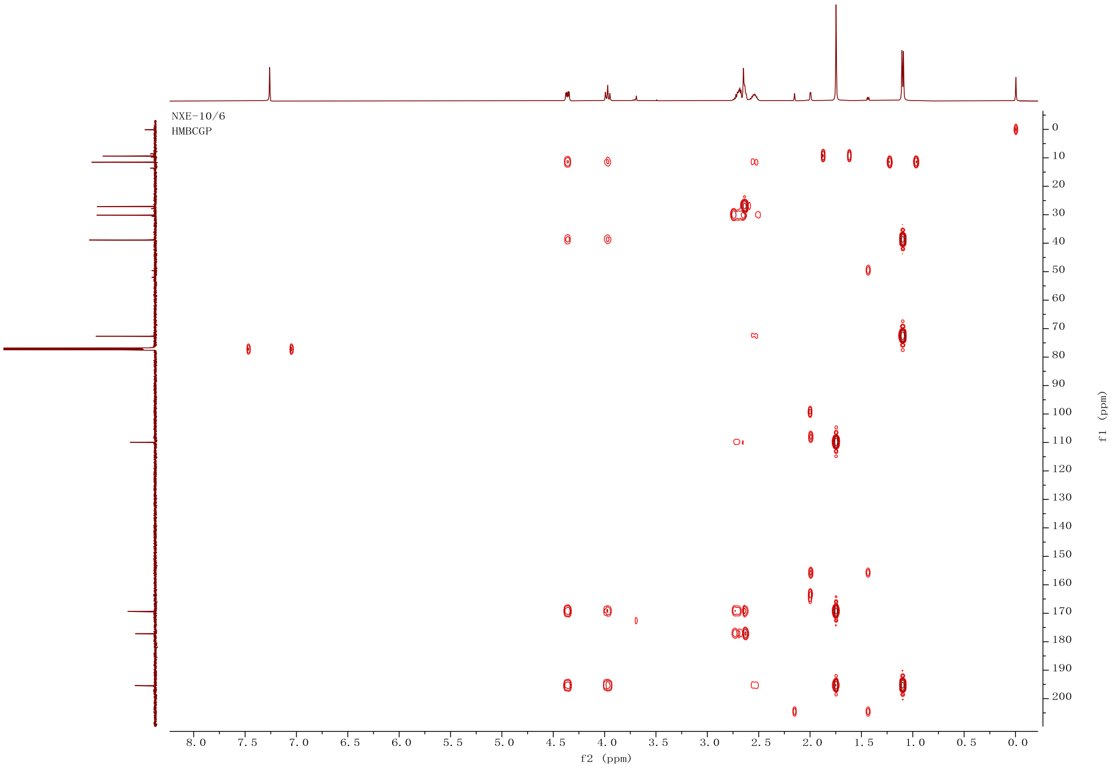

Figure S33. HMBC spectrum (500 MHz, $CDCl_3$) of compound **14**

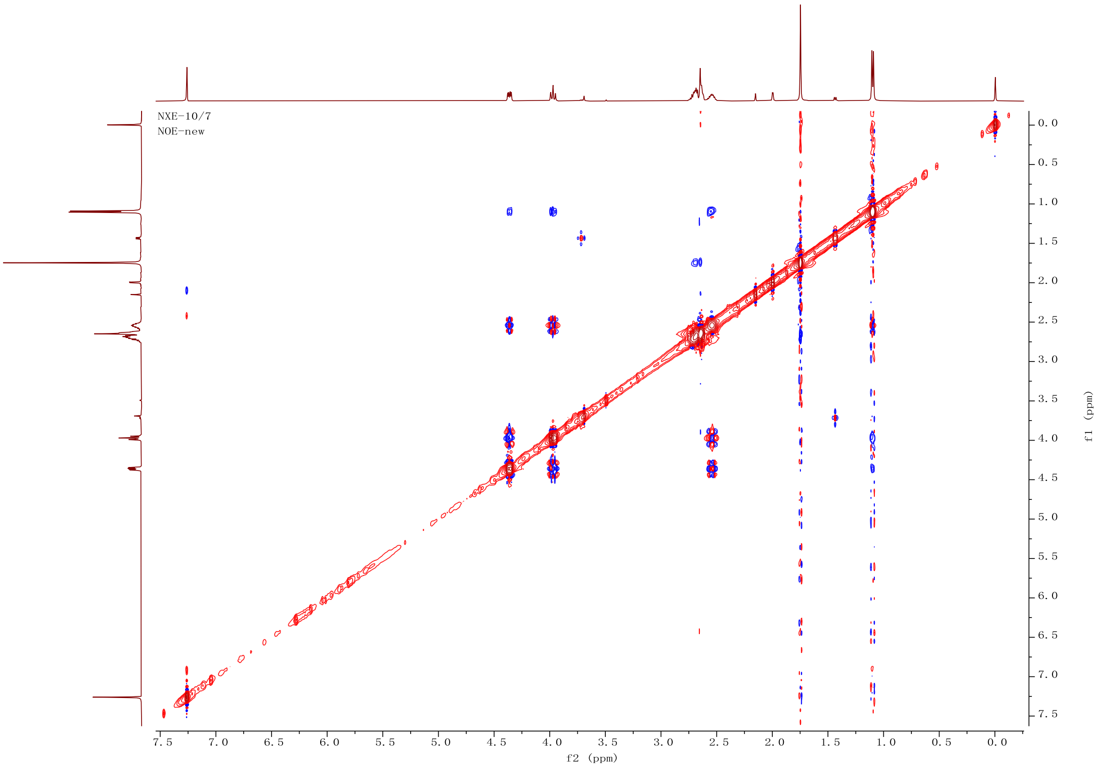

Figure S34. ROESY spectrum (500 MHz, $CDCl_3$) of compound **14**

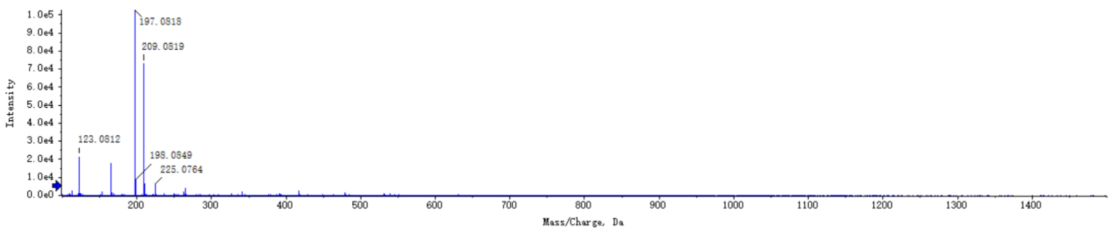

Figure S35. (−)-HRESIMS spectrum of compound **14**

Table S1. Important thermodynamic parameters and Boltzmann distributions of the optimized **1a**, **1b**, **11a**, **11b**, **14a** and **14b** at b3lyp/6-31g* level in gas phase.

Compounds	Conformations	Energy (a.u)	Δ G(kcal/mol)	%
	1	-1339.936146	0	32.10%
	2	-1339.935844	0.19	23.31%
**1a**	3	-1339.935735	0.26	20.77%
	4	-1339.93524	0.57	12.30%
	5	-1339.935179	0.61	11.53%
	1	-1339.936846	0	39.71%
	2	-1339.936409	0.27	25.00%
**1b**	3	-1339.935909	0.59	14.72%
	4	-1339.935757	0.68	12.53%
	5	-1339.935338	0.95	8.04%
	1	-1268.650384	0	47.73%
	2	-1268.649915	0.29	29.04%
**11a**	3	-1268.649097	0.81	12.21%
	4	-1268.648837	0.97	9.27%
	5	-1268.647261	1.96	1.75%
	1	-1268.704888	0	92.02%
**11b**	2	-1268.701973	1.83	4.20%
	3	-1268.701875	1.89	3.78%
	1	-690.095084	0	32.34%
	2	-690.094789	0.19	23.66%
**14a**	3	-690.094502	0.37	17.46%
	4	-690.094413	0.42	15.89%
	5	-690.094035	0.66	10.65%
	1	-690.095034	0	30.05%
	2	-690.094788	0.15	23.15%
**14b**	3	-690.094543	0.31	17.86%
	4	-690.094353	0.43	14.61%
	5	-690.094335	0.44	14.33%

Table S2. The coordinates for the lowest energy conformers of **1a**.

No.	conformer 1			conformer 2			conformer 3			conformer 4			conformer 5		
C	3.855596	-3.02263	1.157288	3.947369	-2.87664	0.888296	4.093279	-2.68215	1.27417	4.015093	-3.04504	0.772909	4.126348	-3.15904	0.438924
C	4.811674	-2.04048	0.883446	4.875583	-1.87735	0.582275	5.10058	-1.85919	0.76246	4.963435	-2.08549	0.40741	5.068913	-2.17902	0.115324
C	4.494247	-0.91084	0.147403	4.510683	-0.74939	-0.13417	4.824475	-0.88403	-0.18197	4.611003	-0.96902	-0.33305	4.69906	-1.00536	-0.52103
C	3.190427	-0.72673	-0.34525	3.185429	-0.58435	-0.57376	3.511749	-0.70003	-0.65079	3.278347	-0.77574	-0.73747	3.354224	-0.77329	-0.85901
C	2.22295	-1.72798	-0.06317	2.246269	-1.60291	-0.25875	2.492904	-1.54186	-0.12969	2.31963	-1.75599	-0.36548	2.401445	-1.77487	-0.53115
C	2.568073	-2.86176	0.683195	2.639404	-2.73454	0.466843	2.796575	-2.51971	0.825938	2.699956	-2.87564	0.385013	2.799522	-2.95248	0.114101
N	2.847586	0.392361	-1.08662	2.796022	0.532169	-1.29536	3.207597	0.270904	-1.59041	2.898837	0.33332	-1.47542	2.957304	0.393121	-1.49186
C	1.548458	0.622986	-1.60379	1.473171	0.744915	-1.75751	1.899528	0.495895	-2.09428	1.568779	0.571001	-1.91072	1.614991	0.669624	-1.86243
C	0.58651	-0.37841	-1.26718	0.541743	-0.27309	-1.3869	0.897046	-0.36577	-1.55212	0.626826	-0.42792	-1.51477	0.680282	-0.35472	-1.51723
C	0.912736	-1.48999	-0.56442	0.913047	-1.38338	-0.70516	1.180504	-1.31922	-0.632	0.984027	-1.51762	-0.79357	1.053926	-1.49844	-0.89443
C	3.855198	1.417181	-1.35363	3.776206	1.572989	-1.60098	4.270464	1.127753	-2.11281	3.900264	1.331875	-1.84509	3.952328	1.413936	-1.81492
O	1.294386	1.629998	-2.27107	1.17624	1.749457	-2.41002	1.679353	1.381117	-2.92523	1.280469	1.575029	-2.56684	1.31211	1.724041	-2.42625
C	-0.86776	-0.38856	-1.61535	-0.92566	-0.30074	-1.67175	-0.56472	-0.37543	-1.87498	-0.84089	-0.45637	-1.8057	-0.79482	-0.35909	-1.76928
C	-1.31399	-1.78848	-0.98423	-1.32482	-1.71205	-1.03422	-1.08443	-1.51875	-0.88597	-1.27235	-1.79469	-1.04534	-1.20924	-1.7539	-1.10735
O	-0.09672	-2.35674	-0.36491	-0.07455	-2.26636	-0.46986	0.126273	-2.04628	-0.21807	-0.02022	-2.36619	-0.50373	0.055032	-2.36484	-0.64165
C	-1.69359	0.835519	-1.12697	-1.74245	0.909925	-1.13624	-1.30672	0.974136	-1.7172	-1.64103	0.797475	-1.37491	-1.58107	0.856787	-1.22016
C	-1.98034	0.867562	0.401039	-1.96663	0.922529	0.402158	-1.42651	1.449087	-0.24486	-1.74041	0.977164	0.163979	-1.6456	0.913113	0.33044
C	-3.59746	-1.34125	0.099482	-3.56763	-1.30365	0.145042	-3.2182	-0.58455	0.212182	-3.42752	-1.1869	0.221839	-3.3354	-1.24763	0.24845
C	-2.25304	-1.36952	0.097412	-2.22419	-1.31506	0.088432	-1.8868	-0.7432	0.10829	-2.09175	-1.26409	0.087142	-2.0029	-1.31481	0.080649
O	-3.45805	1.033149	0.632722	-3.43564	1.067479	0.695046	-2.85752	1.818503	0.038887	-3.16829	1.216956	0.558689	-3.06422	1.121007	0.773746
C	-4.22516	-0.09185	0.657557	-4.18732	-0.06742	0.740191	-3.68555	0.826186	0.466299	-3.95831	0.121813	0.74768	-3.85262	0.014704	0.888957
O	-5.39429	-0.014	0.957776	-5.34401	-0.00708	1.088636	-4.79731	1.099186	0.858257	-5.08501	0.26131	1.164163	-4.97011	0.120019	1.339088
C	-1.78109	-2.77429	-2.02975	-1.8196	-2.69698	-2.06774	-1.7338	-2.67328	-1.61183	-1.86961	-2.82708	-1.97206	-1.82645	-2.71247	-2.09807
N	-1.58795	-0.40134	0.934229	-1.53729	-0.34614	0.90591	-1.08067	0.345002	0.599326	-1.32067	-0.25392	0.768924	-1.21557	-0.36287	0.824635

C	-1.37809	2.041202	1.159589	-1.34813	2.091339	1.155022	-0.62576	2.707445	0.059351	-0.97661	2.189681	0.6672	-0.87066	2.08281	0.9119
C	0.152168	2.108692	1.090538	0.176759	2.186873	1.018896	-0.38136	2.986524	1.552227	-0.90312	2.318652	2.193542	-0.78486	2.105363	2.442506
C	0.699175	3.216438	1.938457	0.739934	3.28145	1.873174	-1.62057	3.016293	2.397334	-0.24558	3.60263	2.600462	-0.02383	3.301429	2.930158
C	1.415788	4.237085	1.47063	1.641385	3.10384	2.837341	-1.79703	2.296692	3.505555	0.908902	3.692457	3.257667	-0.52794	4.258611	3.706217
C	1.969575	5.350694	2.301659	2.21127	4.191834	3.691554	-3.03511	2.308044	4.345033	1.57499	4.972529	3.651555	0.224927	5.458829	4.185517
C	-4.56604	-2.28397	-0.5432	-4.54963	-2.25226	-0.46791	-4.31762	-1.5589	-0.07256	-4.48318	-2.13546	-0.25348	-4.40223	-2.15516	-0.27897
H	-0.99829	-0.43629	-2.7053	-1.10402	-0.34095	-2.75517	-0.72226	-0.7044	-2.91212	-1.01273	-0.58826	-2.88345	-0.99534	-0.40484	-2.84916
H	4.122214	-3.90053	1.736511	4.251292	-3.75312	1.451084	4.328006	-3.43967	2.014719	4.309251	-3.91256	1.354457	4.434147	-4.07144	0.939006
H	5.826564	-2.15724	1.252321	5.906275	-1.97941	0.909366	6.123417	-1.97954	1.10752	6.000212	-2.20984	0.706361	6.114861	-2.33254	0.36457
H	5.262778	-0.17225	-0.04187	5.258881	0.0025	-0.35069	5.632397	-0.26601	-0.55283	5.374994	-0.2482	-0.5954	5.459223	-0.27074	-0.75469
H	1.800409	-3.60313	0.880716	1.892247	-3.48936	0.690364	1.990205	-3.1412	1.201932	1.937893	-3.60066	0.652415	2.041804	-3.69255	0.350891
H	4.222113	1.850051	-0.41581	4.178218	2.006499	-0.67809	5.037998	0.529511	-2.61678	4.355134	1.773943	-0.9514	3.426062	2.239934	-2.29317
H	4.697483	0.997797	-1.91525	4.598442	1.168377	-2.20182	3.813984	1.809599	-2.83012	4.68424	0.884119	-2.46645	4.446984	1.774605	-0.90593
H	3.377418	2.195248	-1.94884	3.261035	2.347143	-2.16935	4.734948	1.705856	-1.30577	3.38922	2.10911	-2.41315	4.707005	1.017566	-2.50386
H	-2.65393	0.842091	-1.65158	-2.72316	0.913841	-1.62176	-2.31271	0.875961	-2.13722	-2.65209	0.734853	-1.78983	-2.60152	0.827591	-1.61503
H	-1.15941	1.739045	-1.43449	-1.22836	1.821053	-1.45557	-0.77612	1.721285	-2.31415	-1.15854	1.670968	-1.82258	-1.10726	1.763035	-1.60798
H	-2.67475	-2.4012	-2.53701	-2.00525	-3.67828	-1.62339	-2.00131	-3.47671	-0.92076	-2.08041	-3.76096	-1.44467	-1.12738	-2.86024	-2.92661
H	-0.98902	-2.90294	-2.77377	-2.73807	-2.33337	-2.53606	-2.63173	-2.3369	-2.1375	-2.79269	-2.45073	-2.4214	-2.0272	-3.68402	-1.63917
H	-1.99875	-3.74933	-1.58617	-1.05622	-2.80858	-2.84382	-1.02945	-3.07183	-2.34807	-1.15456	-3.0367	-2.77303	-2.75806	-2.30488	-2.50028
H	-1.88067	-0.48783	1.903205	-1.78689	-0.44456	1.885738	-1.31304	0.566721	1.563917	-1.52294	-0.24946	1.76357	-1.39654	-0.44082	1.820252
H	-1.81559	2.96323	0.759032	-1.825	3.011637	0.795793	0.342377	2.607405	-0.44643	0.035722	2.134198	0.248078	0.135877	2.047011	0.474807
H	-1.7076	1.969634	2.205252	-1.62108	1.992252	2.213922	-1.14985	3.547629	-0.41211	-1.45373	3.079274	0.236996	-1.34372	3.005596	0.55509
H	0.563699	1.144068	1.420464	0.6347	1.227706	1.288944	0.323618	2.246796	1.953193	-1.92282	2.278308	2.60228	-1.79361	2.103033	2.872038
H	0.474998	2.260482	0.055273	0.43262	2.38449	-0.0306	0.127289	3.959079	1.615914	-0.33701	1.478176	2.615324	-0.27003	1.196148	2.788143
H	0.481187	3.167731	3.008249	0.370332	4.290428	1.674263	-2.4293	3.662636	2.0538	-0.76302	4.51987	2.310185	1.015033	3.378948	2.600385
H	1.622461	4.27727	0.398616	2.005215	2.092163	3.030763	-0.9856	1.644948	3.842271	1.42155	2.770509	3.540746	-1.56846	4.176762	4.02698

H	1.711548	5.228227	3.357891	3.301751	4.248659	3.584797	-3.47163	1.304063	4.415281	2.577723	5.046518	3.212573	0.254319	5.494421	5.281634
H	3.062538	5.400452	2.219485	1.791171	5.167384	3.42914	-2.81608	2.630598	5.370584	0.994734	5.841341	3.326928	1.254434	5.459161	3.815184
H	1.585693	6.321776	1.965275	2.009545	4.006325	4.753871	-3.79339	2.974775	3.925171	1.703016	5.03291	4.739442	-0.25953	6.385724	3.854378
H	-5.00737	-1.85432	-1.45156	-4.09236	-3.20379	-0.73544	-3.96105	-2.58775	-0.09809	-5.24532	-2.22395	0.527392	-4.00544	-3.12218	-0.58493
H	-4.11044	-3.24343	-0.78387	-5.35106	-2.43054	0.256251	-5.0749	-1.46237	0.712314	-4.99962	-1.75073	-1.14213	-5.14743	-2.3069	0.50853
H	-5.39729	-2.4457	0.150584	-5.03099	-1.81968	-1.35425	-4.82702	-1.32897	-1.01717	-4.08053	-3.12361	-0.47184	-4.93745	-1.70055	-1.12235

Table S3. The coordinates for the lowest energy conformers of **1b**.

No.	conformer 1			conformer 2			conformer 3			conformer 4			conformer 5		
C	-4.31112	2.282909	-1.03667	-4.02745	2.219807	-1.34805	-3.95578	2.808729	-0.91519	-4.12067	2.642114	-1.28055	-4.02224	2.292797	-1.35966
C	-5.19362	1.359263	-0.46965	-4.89804	1.209437	-0.92936	-4.86576	1.780474	-0.6537	-5.1194	1.822627	-0.74692	-4.93049	1.345735	-0.87889
C	-4.75931	0.417835	0.449232	-4.49413	0.237052	-0.02925	-4.48745	0.641685	0.038275	-4.82996	0.862802	0.209172	-4.53578	0.34967	-0.00014
C	-3.40811	0.371682	0.835216	-3.18626	0.245266	0.487045	-3.16672	0.494694	0.497372	-3.51201	0.691409	0.66801	-3.19976	0.26963	0.429438
C	-2.51698	1.316431	0.259255	-2.30736	1.276842	0.060749	-2.24611	1.542891	0.227943	-2.50189	1.529583	0.124462	-2.27957	1.228981	-0.06997
C	-2.97946	2.257098	-0.66954	-2.73864	2.248667	-0.85111	-2.6526	2.684968	-0.47356	-2.819	2.491827	-0.84257	-2.70292	2.229844	-0.95282
N	-2.94643	-0.56328	1.746865	-2.75173	-0.72386	1.375133	-2.76439	-0.63234	1.19502	-3.19465	-0.26374	1.619358	-2.77862	-0.71669	1.306095
C	-1.59403	-0.64657	2.1728	-1.43594	-0.76983	1.909412	-1.44565	-0.82731	1.676171	-1.88082	-0.47546	2.113951	-1.43634	-0.8553	1.747397
C	-0.72241	0.314434	1.57405	-0.57159	0.2715	1.450528	-0.53176	0.221704	1.35125	-0.88742	0.382291	1.549382	-0.53225	0.117951	1.221552
C	-1.15989	1.232251	0.678446	-0.98621	1.229675	0.586169	-0.91648	1.341879	0.693518	-1.18383	1.320635	0.617872	-0.93506	1.098789	0.377801
C	-3.87809	-1.53154	2.321404	-3.66805	-1.78387	1.792093	-3.72515	-1.70373	1.452934	-4.24883	-1.11617	2.166049	-3.73871	-1.70205	1.799589
O	-1.23303	-1.50021	2.986916	-1.09571	-1.66318	2.688823	-1.13719	-1.84245	2.306567	-1.64883	-1.34657	2.956524	-1.10926	-1.75867	2.521498
C	0.748515	0.471877	1.801541	0.880768	0.454469	1.767917	0.928862	0.275186	1.665657	0.57678	0.404838	1.860094	0.9196	0.272886	1.548167
C	1.082853	1.668747	0.796625	1.234749	1.712072	0.845551	1.306292	1.712794	1.075516	1.081872	1.53693	0.850737	1.26927	1.591444	0.715645
O	-0.21492	2.064967	0.204806	-0.04692	2.123914	0.229236	0.054158	2.253807	0.502279	-0.13717	2.046975	0.184169	0.02377	1.966197	0.01062
C	1.602614	-0.80177	1.580316	1.782639	-0.77882	1.510684	1.784062	-0.90088	1.113184	1.325866	-0.94233	1.715993	1.832876	-0.95414	1.266367

C	1.693215	-1.25075	0.09478	1.893222	-1.15538	0.006881	2.037829	-0.86441	-0.42014	1.437852	-1.4376	0.249805	2.205155	-1.17806	-0.22432
C	3.234957	0.963641	-0.42299	3.432725	1.092969	-0.34582	3.57983	1.391799	-0.07202	3.213562	0.600328	-0.24909	3.604399	1.205746	-0.26116
C	1.901722	0.982711	-0.24829	2.095497	1.094593	-0.21101	2.235736	1.369086	-0.04001	1.881928	0.75222	-0.13812	2.26466	1.095492	-0.2761
O	3.131462	-1.46779	-0.28257	3.342526	-1.34544	-0.36528	3.515622	-0.96591	-0.6879	2.869206	-1.8022	-0.03878	3.714017	-1.2198	-0.36955
C	3.834253	-0.38608	-0.71978	4.051522	-0.234	-0.70512	4.239914	0.187347	-0.68871	3.688227	-0.81103	-0.48559	4.367457	-0.04925	-0.58617
O	4.951356	-0.53564	-1.15974	5.184559	-0.34605	-1.11421	5.403932	0.164308	-1.01676	4.799245	-1.08267	-0.88064	5.548165	-0.05729	-0.85232
C	1.64201	2.885677	1.495106	1.753625	2.893218	1.630138	1.757324	2.678678	2.146451	1.728293	2.7063	1.555204	1.630072	2.755932	1.609517
N	1.180632	-0.17717	-0.70067	1.394189	-0.04578	-0.7386	1.587578	0.407398	-0.89673	1.079127	-0.34789	-0.60754	1.716743	-0.05478	-0.9538
C	1.014136	-2.58911	-0.16084	1.237557	-2.48893	-0.31831	1.463188	-2.0266	-1.21688	0.643095	-2.70526	-0.03079	1.805913	-2.53029	-0.79781
C	1.19373	-3.18245	-1.56764	1.170495	-2.85132	-1.80427	-0.06122	-2.16306	-1.11482	0.391077	-3.00803	-1.51778	0.304754	-2.82614	-0.87966
C	0.602062	-2.36791	-2.67913	0.027375	-2.28183	-2.60342	-0.57714	-3.25883	-1.99692	1.624792	-3.04146	-2.37078	-0.41275	-2.1084	-1.98745
C	1.284464	-1.91779	-3.73329	-0.99749	-1.56042	-2.15174	-1.46084	-3.0902	-2.97893	1.78813	-2.33765	-3.49109	-1.67585	-1.6939	-1.9358
C	0.702383	-1.11185	-4.85119	-2.12178	-1.05027	-2.99595	-1.98294	-4.17982	-3.86112	3.020277	-2.353	-4.33919	-2.41384	-1.02711	-3.05149
C	4.239766	2.046264	-0.18094	4.419883	2.166252	-0.01028	4.527383	2.346377	0.584216	4.308444	1.585859	0.013595	4.456485	2.337933	0.220731
H	0.940975	0.807427	2.830613	1.013844	0.735729	2.822215	1.085166	0.28874	2.753136	0.740489	0.749475	2.891172	1.034875	0.499105	2.617449
H	-4.66871	3.012048	-1.75625	-4.36055	2.971911	-2.05566	-4.26996	3.693315	-1.45935	-4.36579	3.387616	-2.02987	-4.34909	3.066361	-2.04676
H	-6.24291	1.372629	-0.74992	-5.91333	1.178115	-1.3139	-5.8926	1.868068	-0.9967	-6.14602	1.933514	-1.08379	-5.96873	1.383845	-1.19546
H	-5.47405	-0.28088	0.865333	-5.1978	-0.53015	0.268301	-5.2217	-0.1327	0.220651	-5.63158	0.246941	0.596919	-5.27004	-0.36481	0.350014
H	-2.26726	2.959544	-1.09053	-2.03514	3.016134	-1.15767	-1.91948	3.462688	-0.66237	-2.01902	3.111104	-1.23549	-1.9696	2.9449	-1.31163
H	-3.30805	-2.16781	2.998278	-3.9982	-2.37291	0.928714	-3.20162	-2.48249	2.007159	-3.78241	-1.78363	2.890436	-4.55046	-1.21233	2.349006
H	-4.67045	-1.02368	2.883119	-3.12462	-2.4282	2.482915	-4.56642	-1.33588	2.051055	-5.01419	-0.51267	2.667039	-4.16153	-2.28178	0.970359
H	-4.33021	-2.14792	1.536006	-4.54322	-1.36291	2.299752	-4.10142	-2.11856	0.510833	-4.71834	-1.70992	1.373397	-3.19991	-2.37138	2.470085
H	2.615407	-0.62244	1.954756	2.784128	-0.57456	1.901922	2.75533	-0.89636	1.617338	2.33448	-0.83121	2.126301	2.761303	-0.84245	1.834691
H	1.166733	-1.59932	2.188718	1.373281	-1.61902	2.078459	1.285621	-1.83215	1.397299	0.804971	-1.68418	2.327935	1.322439	-1.83581	1.66514
H	0.94179	3.201648	2.274254	1.905035	3.762322	0.984807	1.92592	3.677077	1.734609	2.632371	2.384364	2.079399	2.540068	2.541287	2.176314
H	2.601008	2.651995	1.965627	1.021646	3.157909	2.398887	0.977778	2.748025	2.911216	1.984951	3.501507	0.850627	1.772727	3.671718	1.030352

H	1.777402	3.716077	0.7973	2.697589	2.642374	2.121916	2.676256	2.324047	2.620678	1.026137	3.110376	2.290525	0.812441	2.921316	2.318068
H	1.32123	-0.36261	-1.6903	1.52067	-0.16703	-1.73817	1.85288	0.538671	-1.86854	1.305739	-0.58199	-1.5706	2.008192	-0.09007	-1.92574
H	1.411824	-3.30163	0.571503	1.819241	-3.24905	0.215704	1.755049	-1.8933	-2.26692	1.175327	-3.53508	0.449806	2.253495	-2.61773	-1.79711
H	-0.04938	-2.44944	0.074127	0.239663	-2.48829	0.135882	1.95489	-2.94447	-0.87162	-0.32254	-2.6036	0.479464	2.302173	-3.28562	-0.17743
H	2.259218	-3.35098	-1.75715	2.125143	-2.58692	-2.2831	-0.33354	-2.38298	-0.07402	-0.11084	-3.98507	-1.56393	0.217509	-3.90889	-1.0559
H	0.71026	-4.17019	-1.55868	1.118987	-3.94645	-1.89009	-0.53884	-1.21228	-1.3804	-0.32197	-2.27946	-1.9249	-0.19078	-2.64075	0.081583
H	-0.4633	-2.13555	-2.6	0.05299	-2.53072	-3.66699	-0.18639	-4.26091	-1.80365	2.440558	-3.67668	-2.02323	0.148044	-1.97068	-2.9157
H	2.35122	-2.14425	-3.7977	-1.03918	-1.2881	-1.09777	-1.84616	-2.0854	-3.16646	0.969625	-1.69693	-3.83182	-2.23241	-1.84409	-1.00938
H	1.216724	-0.14782	-4.95232	-2.18781	0.043843	-2.9387	-1.54349	-5.14795	-3.60294	3.786105	-3.00833	-3.91509	-1.77933	-0.90727	-3.93525
H	0.820136	-1.62925	-5.81129	-2.00451	-1.33883	-4.04513	-3.07368	-4.26626	-3.78	2.796134	-2.69219	-5.35825	-3.30012	-1.60543	-3.34212
H	-0.36307	-0.91885	-4.69387	-3.08618	-1.43266	-2.63795	-1.76235	-3.97362	-4.91584	3.449368	-1.34723	-4.42753	-2.77302	-0.03606	-2.7465
H	4.813828	1.867215	0.737164	4.971073	1.934305	0.910056	5.003458	1.901516	1.467335	4.826382	1.372976	0.957567	4.9113	2.115986	1.194432
H	4.965035	2.031833	-1.00088	5.167222	2.209144	-0.80909	5.336953	2.56325	-0.12008	5.06071	1.483257	-0.77534	5.287618	2.46173	-0.48104
H	3.778911	3.031784	-0.13016	3.948364	3.143041	0.089908	4.042598	3.279217	0.868639	3.945096	2.612561	0.027044	3.902518	3.273147	0.286464

Table S4. The coordinates for the lowest energy conformers of **11a**.

No.	conformer 1			conformer 2			conformer 3			conformer 4			conformer 5		
C	-3.31886	2.195767	0.28511	-3.48635	2.18414	-0.19128	-3.31041	2.147581	0.44664	-3.50329	2.155193	-0.07554	-3.31307	2.227212	0.227286
C	-4.63166	1.986108	0.554479	-4.75828	1.941722	0.213462	-4.63151	1.952014	0.655884	-4.79018	1.908851	0.25665	-4.62469	2.024487	0.50711
C	-5.59963	0.773924	0.507681	-5.60981	0.681461	0.523802	-5.6315	0.783887	0.487709	-5.67729	0.656417	0.450375	-5.58745	0.807141	0.509754
C	-5.37117	-0.68909	-0.0292	-5.26712	-0.84546	0.345254	-5.41224	-0.66279	-0.09852	-5.3389	-0.86861	0.240146	-5.3616	-0.66882	0.007649
C	-3.89079	-0.96324	-0.22504	-3.77806	-1.03644	0.119423	-3.92634	-0.94216	-0.22898	-3.83935	-1.04531	0.090621	-3.88213	-0.94872	-0.1885
C	-3.34856	0.191175	-1.01923	-3.38903	-0.07553	-0.96805	-3.33142	0.219893	-0.97385	-3.3901	-0.06736	-0.95884	-3.34434	0.184066	-1.0159
C	-2.45455	1.00404	-0.0635	-2.5295	1.017375	-0.30584	-2.44331	0.972891	0.037668	-2.54204	0.992206	-0.22659	-2.4495	1.025059	-0.08518
C	-0.75491	-0.67407	-0.82302	-0.71747	-0.65273	-0.76078	-0.73983	-0.65629	-0.76804	-0.71895	-0.63929	-0.703	-0.75023	-0.67352	-0.80329

C	-1.06118	0.87167	-0.68292	-1.16419	0.848746	-0.97703	-1.04789	0.885199	-0.58644	-1.1697	0.865586	-0.89344	-1.05675	0.875342	-0.70181
C	-3.048	-2.13178	-0.66128	-2.84867	-2.20392	-0.08072	-3.06501	-2.10446	-0.63492	-2.88496	-2.19571	-0.06297	-3.04084	-2.12882	-0.59466
C	-1.55754	-1.7806	-0.80918	-1.40894	-1.78162	-0.41933	-1.55493	-1.7575	-0.7497	-1.42135	-1.76324	-0.3558	-1.55117	-1.78037	-0.75747
O	-3.39948	-3.28069	-0.8385	-3.097	-3.38557	0.049847	-3.39579	-3.25532	-0.82921	-3.10879	-3.38165	0.059161	-3.3918	-3.2825	-0.73877
C	1.446654	1.358542	-0.33667	1.299492	1.609156	-0.94953	1.463651	1.356978	-0.24137	1.291361	1.625053	-0.8486	1.451221	1.371783	-0.37039
C	0.010136	1.686886	0.064091	-0.13842	1.904221	-0.52813	0.029155	1.669044	0.186838	-0.14448	1.904441	-0.40502	0.015253	1.709648	0.023561
C	0.700114	-0.86651	-1.19285	0.728493	-0.81423	-1.17917	0.709764	-0.84685	-1.15152	0.724867	-0.79849	-1.12278	0.703597	-0.87307	-1.17395
C	1.695138	-0.14304	-0.24995	1.702842	0.201491	-0.52556	1.725156	-0.14562	-0.21216	1.709837	0.207985	-0.46964	1.699369	-0.12763	-0.24896
O	-6.70855	1.020598	0.94551	-6.71737	0.938595	0.958834	-6.76236	1.044033	0.856337	-6.81406	0.905922	0.807887	-6.69075	1.061671	0.957168
H	-3.47041	-0.88333	0.788847	-3.32115	-0.67297	1.051924	-3.58394	-0.85693	0.814903	-3.46552	-0.66712	1.055641	-3.4567	-0.84138	0.820823
C	1.541298	-0.64349	1.190881	1.692298	0.058884	0.999677	1.63631	-0.68993	1.216606	1.753083	0.031468	1.051013	1.540835	-0.5959	1.202332
H	-1.09987	1.23601	-1.72277	-1.29201	0.94183	-2.06826	-1.08222	1.282695	-1.61422	-1.28191	0.986743	-1.9836	-1.09622	1.212973	-1.7506
H	0.814507	-0.3591	-2.16971	0.740655	-0.56563	-2.25746	0.809088	-0.3145	-2.11657	0.724549	-0.53246	-2.19691	0.814273	-0.38767	-2.16233
O	-2.2071	0.426948	1.263417	-2.15784	0.808928	1.098633	-2.2857	0.375527	1.361821	-2.27703	0.770818	1.193311	-2.20193	0.487042	1.258087
C	1.287862	-2.27493	-1.34822	1.435626	-2.16711	-1.01648	1.291681	-2.2493	-1.35725	1.432229	-2.15363	-0.995	1.293815	-2.28347	-1.29877
C	2.824419	-2.07118	-1.26455	2.94912	-1.83305	-1.07562	2.82926	-2.03896	-1.32011	2.944151	-1.8153	-1.08399	2.830374	-2.07097	-1.24205
C	3.04187	-0.58038	-0.90301	3.046843	-0.28974	-1.15263	3.054871	-0.55919	-0.91656	3.038389	-0.27006	-1.13976	3.044813	-0.57415	-0.89644
C	4.282406	-0.23206	-0.11568	4.298791	0.366345	-0.6206	4.3167	-0.23525	-0.15042	4.304756	0.378915	-0.63146	4.292095	-0.21549	-0.1244
C	4.803176	-1.21209	0.889841	4.789842	1.526938	-1.43339	4.880779	-1.25741	0.787527	4.773993	1.549264	-1.44234	4.728073	-1.10704	1.005092
C	4.861566	0.961369	-0.36613	4.901058	-0.08273	0.49743	4.873132	0.976402	-0.35864	4.936355	-0.0862	0.463257	4.940783	0.908468	-0.4977
C	6.045078	1.507767	0.301369	6.105323	0.481876	1.112631	6.070254	1.507128	0.299729	6.156431	0.469567	1.057925	6.141973	1.528765	0.06383
O	6.773406	0.969341	1.112425	6.830223	1.364279	0.695816	6.830584	0.939097	1.059106	6.867398	1.362131	0.640121	6.565193	2.608404	-0.30207
O	6.276272	2.7885	-0.12214	6.361572	-0.14537	2.30129	6.2658	2.81171	-0.06111	6.442988	-0.18062	2.226404	6.781274	0.805215	1.0291
H	-2.89046	3.177439	0.484465	-3.14407	3.216079	-0.2617	-2.86822	3.09722	0.742269	-3.14494	3.182786	-0.0577	-2.88552	3.215781	0.391023
H	-5.19775	2.828926	0.946605	-5.38171	2.806571	0.432399	-5.18908	2.783285	1.08309	-5.41015	2.771647	0.492524	-5.1916	2.8785	0.872907
H	-5.86879	-1.34873	0.687623	-5.66367	-1.34421	1.234437	-5.94704	-1.34066	0.572807	-5.7766	-1.39145	1.095314	-5.85636	-1.31001	0.74286

H	-5.93858	-0.74717	-0.96828	-5.87034	-1.18255	-0.50896	-5.9376	-0.6768	-1.06285	-5.89888	-1.17819	-0.65246	-5.93302	-0.74987	-0.92738
H	-4.14588	0.811392	-1.4219	-4.26094	0.359748	-1.4506	-4.0972	0.881657	-1.37022	-4.23205	0.40203	-1.46095	-4.14424	0.790702	-1.43371
H	-2.78636	-0.1433	-1.88874	-2.84302	-0.5701	-1.769	-2.76346	-0.10491	-1.84441	-2.82653	-0.55523	-1.75283	-2.78301	-0.17294	-1.87694
H	-1.04096	-2.71581	-1.00901	-0.81613	-2.69251	-0.42304	-1.04455	-2.69721	-0.94726	-0.83036	-2.67589	-0.34263	-1.03409	-2.71983	-0.9351
H	2.146153	1.907901	0.306385	1.966646	2.358392	-0.50385	2.169243	1.888297	0.409858	1.96181	2.365058	-0.39298	2.152406	1.936281	0.257799
H	1.634339	1.688662	-1.36939	1.399183	1.693137	-2.04236	1.634383	1.723435	-1.26452	1.374209	1.73903	-1.93992	1.637964	1.678015	-1.41047
H	-0.19178	2.748894	-0.12754	-0.45395	2.869119	-0.94642	-0.1747	2.736623	0.037154	-0.4647	2.882794	-0.78468	-0.18737	2.766312	-0.19472
H	-0.11685	1.534818	1.138377	-0.18879	2.003112	0.55876	-0.09095	1.488593	1.259146	-0.1905	1.974759	0.685837	-0.11091	1.584576	1.101404
H	0.516656	-0.49684	1.544894	2.325584	0.821231	1.46413	2.360697	-0.18592	1.865389	2.404791	0.781125	1.509627	0.515306	-0.43907	1.549337
H	1.756638	-1.71213	1.278492	0.676591	0.160176	1.393785	0.642954	-0.52654	1.644615	0.757847	0.133156	1.494428	1.752321	-1.66273	1.314194
H	2.218115	-0.10373	1.862465	2.064355	-0.91841	1.319877	1.828497	-1.76542	1.262446	2.128596	-0.95432	1.338079	2.215412	-0.04349	1.865354
H	-2.84381	0.810017	1.879278	-2.79863	1.274773	1.649804	-1.6308	-0.33769	1.289968	-1.55856	0.121512	1.263767	-2.8384	0.888678	1.862263
H	0.97778	-2.74795	-2.28469	1.133625	-2.88335	-1.78643	0.952356	-2.70099	-2.2941	1.113168	-2.85702	-1.76984	0.974304	-2.78306	-2.21812
H	0.950617	-2.92449	-0.53384	1.188206	-2.61285	-0.04777	0.982483	-2.91934	-0.54799	1.207464	-2.61731	-0.0288	0.969318	-2.91176	-0.46274
H	3.254214	-2.73546	-0.51046	3.461381	-2.22617	-0.19426	3.288905	-2.72616	-0.60548	3.477554	-2.22037	-0.22079	3.280834	-2.73087	-0.49567
H	3.323498	-2.30826	-2.20914	3.440371	-2.28303	-1.94361	3.294457	-2.24155	-2.28956	3.415327	-2.25152	-1.96971	3.312796	-2.30716	-2.19537
H	3.090583	-0.01389	-1.84348	2.981198	-0.01159	-2.2158	3.078996	0.038863	-1.83814	2.94403	0.025365	-2.19588	3.090168	-0.0236	-1.84582
H	5.460686	-1.93223	0.383676	5.476878	2.170007	-0.88643	5.512815	-0.80239	1.54886	5.2578	1.172193	-2.3546	4.986219	-0.53047	1.895082
H	5.400194	-0.72426	1.659186	5.324202	1.139152	-2.3123	4.092943	-1.85142	1.255661	3.91999	2.149183	-1.77404	3.958142	-1.83225	1.264908
H	3.993491	-1.78605	1.344639	3.944072	2.10605	-1.81807	5.512676	-1.9559	0.221868	5.495468	2.170675	-0.91534	5.63098	-1.66047	0.722041
H	4.431407	1.605963	-1.12826	4.486213	-0.9316	1.029828	4.412254	1.651944	-1.07473	4.537577	-0.94453	0.992969	4.551337	1.486336	-1.33159
H	7.072406	3.082446	0.34704	7.168524	0.264905	2.648951	7.070649	3.097014	0.398638	7.257161	0.224148	2.563767	7.547607	1.342249	1.285567

Table S5. The coordinates for the lowest energy conformers of **11b**.

No.	conformer 1			conformer 2			conformer 3		
C	-3.49722	2.063411	-0.42869	-3.25818	2.189452	0.002971	-3.48163	2.064329	-0.46208
C	-4.75023	1.970635	0.036076	-4.53972	2.139143	0.389999	-4.73477	1.986318	0.004986
C	-5.45054	0.793376	0.665616	-5.39106	0.93946	0.71845	-5.44082	0.823799	0.655303
C	-4.9747	-0.67847	0.702707	-5.06795	-0.55088	0.453277	-4.97353	-0.64993	0.71608
C	-3.48667	-0.9884	1.014724	-3.63946	-1.08393	0.740796	-3.48697	-0.96312	1.031935
C	-2.66525	0.267841	1.046187	-2.70023	0.038071	1.077185	-2.65846	0.288846	1.042084
C	-2.64508	0.826089	-0.41611	-2.53787	0.890719	-0.22495	-2.63652	0.822584	-0.42921
C	-0.68002	-0.6361	-0.7974	-0.71218	-0.66424	-0.85186	-0.67971	-0.65633	-0.78866
C	-1.19358	0.814208	-0.88111	-1.07078	0.8185	-0.63044	-1.18592	0.794705	-0.89563
C	-2.8064	-1.96112	0.04229	-3.00478	-1.89538	-0.39654	-2.81309	-1.95494	0.074471
C	-1.37022	-1.73477	-0.4101	-1.52927	-1.7378	-0.73829	-1.37536	-1.74433	-0.38153
O	-3.34583	-2.99528	-0.30563	-3.62407	-2.76553	-0.98053	-3.35871	-2.99091	-0.25774
C	1.19613	1.654054	-0.594	1.370558	1.303631	-0.07775	1.208152	1.625125	-0.63294
C	-0.26624	1.80406	-0.15317	-0.09274	1.523408	0.328684	-0.25043	1.788838	-0.18503
C	0.737227	-0.71923	-1.28504	0.716209	-0.79455	-1.29417	0.736829	-0.75646	-1.27604
C	1.678503	0.208675	-0.46047	1.686838	-0.18248	-0.24305	1.68552	0.180629	-0.47152
O	-6.55135	1.029361	1.143102	-6.49139	1.1881	1.190669	-6.53919	1.074474	1.130883
H	-3.43431	-1.48743	1.993646	-3.69471	-1.78373	1.587779	-3.43641	-1.44652	2.018753
C	1.72452	-0.21103	1.011765	1.586145	-0.90858	1.102546	1.741148	-0.21	1.009007
H	-1.16759	1.089472	-1.95059	-0.95612	1.312919	-1.6116	-1.1598	1.053217	-1.96936
H	0.740719	-0.29406	-2.30458	0.825679	-0.15369	-2.18709	0.742238	-0.35096	-2.30375
O	-3.37307	-0.00169	-1.37458	-3.29001	0.371744	-1.36528	-3.37031	-0.01687	-1.3732
C	1.483058	-2.05286	-1.34013	1.335792	-2.15177	-1.62912	1.47133	-2.09555	-1.30545
C	2.982525	-1.64911	-1.3833	2.86772	-1.89857	-1.54382	2.972984	-1.70537	-1.36579

C	3.027624	-0.1103	-1.18334	3.040117	-0.46837	-0.96211	3.037349	-0.16627	-1.1801
C	4.265274	0.486883	-0.55539	4.283214	-0.19367	-0.14561	4.259287	0.438718	-0.52881
C	4.670743	1.814418	-1.12284	4.823379	-1.28192	0.737297	4.538921	1.870094	-0.91131
C	4.925558	-0.14755	0.431989	4.827444	1.03551	-0.2665	5.003333	-0.26736	0.344673
C	6.122448	0.341884	1.12374	5.996049	1.623599	0.393121	6.180735	0.149647	1.109411
O	6.786816	1.33135	0.88609	6.277622	2.803025	0.308741	6.747802	-0.57795	1.900886
O	6.449999	-0.50661	2.14513	6.777596	0.754154	1.096698	6.613564	1.423437	0.878387
H	-3.13166	2.991762	-0.86267	-2.77559	3.142418	-0.20388	-3.11136	2.982921	-0.91255
H	-5.40787	2.835586	-0.00176	-5.10391	3.059602	0.518173	-5.38775	2.854043	-0.04736
H	-5.23822	-1.10903	-0.26969	-5.3199	-0.73416	-0.59722	-5.24023	-1.09496	-0.24892
H	-5.6295	-1.14882	1.439735	-5.8078	-1.09007	1.049204	-5.63033	-1.10435	1.461254
H	-1.65456	0.040075	1.390756	-1.73946	-0.36744	1.400626	-1.64877	0.061395	1.389772
H	-3.07908	1.005622	1.734855	-3.08053	0.651224	1.895247	-3.06773	1.040049	1.718895
H	-0.87093	-2.69448	-0.53005	-1.12174	-2.69792	-1.04978	-0.88119	-2.70868	-0.48385
H	1.824754	2.32893	0.000461	2.037193	1.761587	0.663713	1.842214	2.311827	-0.05924
H	1.28962	1.964429	-1.64551	1.559742	1.813834	-1.0339	1.293881	1.913399	-1.69136
H	-0.60897	2.824082	-0.36488	-0.31589	2.596956	0.327351	-0.58904	2.808004	-0.40757
H	-0.34676	1.671856	0.931206	-0.25424	1.177593	1.355338	-0.32466	1.67021	0.901442
H	0.726454	-0.20741	1.459016	0.57519	-0.84991	1.514766	0.743957	-0.22632	1.45807
H	2.125951	-1.21977	1.1411	1.827195	-1.9715	1.017491	2.176074	-1.20232	1.156649
H	2.360662	0.470962	1.583869	2.272698	-0.46141	1.829391	2.359044	0.500254	1.567392
H	-2.74526	-0.55116	-1.8614	-2.6912	-0.11633	-1.94496	-2.74602	-0.57625	-1.85325
H	1.183047	-2.65497	-2.20297	1.024091	-2.51407	-2.61325	1.161883	-2.71578	-2.15197
H	1.276469	-2.64843	-0.44509	1.033341	-2.90881	-0.89828	1.265967	-2.66803	-0.39508
H	3.5427	-2.17515	-0.60717	3.347001	-2.65488	-0.91689	3.529022	-2.23979	-0.59367
H	3.452848	-1.91344	-2.33502	3.346749	-1.96169	-2.52545	3.431708	-1.981	-2.3199

H	2.935499	0.353061	-2.17762	3.060323	0.229962	-1.81007	2.96502	0.28712	-2.18053
H	5.175442	1.646965	-2.08481	5.623807	-1.8199	0.212967	5.53976	1.969359	-1.34153
H	3.784856	2.41975	-1.34138	5.258233	-0.88655	1.654579	3.811518	2.228163	-1.64264
H	5.356535	2.360316	-0.4778	4.055002	-2.01282	0.990763	4.516535	2.532382	-0.04068
H	4.571687	-1.10887	0.788465	4.357247	1.749651	-0.93781	4.743093	-1.29429	0.57279
H	7.247452	-0.13577	2.553878	7.501004	1.292057	1.45592	7.390972	1.53634	1.447872

Table S6. The coordinates for the lowest energy conformers of **14a**.

No.	conformer 1			conformer 2			conformer 3			conformer 4			conformer 5		
C	2.890667	0.602791	-0.20899	2.41893	0.606074	0.115086	2.566773	0.499263	0.220853	2.653043	0.372165	-0.67976	2.243761	-0.08101	-0.81865
C	1.85074	1.482377	0.452799	1.145373	1.095853	0.770656	1.435263	1.385624	0.694069	1.972956	1.562394	-0.0381	1.811581	1.361559	-0.66657
O	0.562555	1.363486	-0.18429	0.004022	0.948631	-0.09994	0.37104	1.454011	-0.27527	0.539967	1.41054	-0.00656	0.401042	1.475047	-0.3961
C	0.131385	0.095747	-0.38905	-0.13988	-0.27965	-0.64338	-0.06275	0.257318	-0.74326	0.089576	0.223157	0.46391	-0.07084	0.665923	0.584648
C	0.929021	-1.00288	-0.30066	0.852059	-1.20849	-0.70953	0.619561	-0.91067	-0.60052	0.867444	-0.88408	0.614206	0.606222	-0.39779	1.092936
C	2.379412	-0.83813	-0.20225	2.215398	-0.84219	-0.33562	1.996205	-0.88304	-0.10211	2.224968	-0.89302	0.064757	1.840535	-0.84402	0.444012
O	3.151375	-1.78849	-0.18526	3.164723	-1.61109	-0.43124	2.689067	-1.88844	-0.00865	2.959153	-1.87139	0.122056	2.483955	-1.8124	0.828219
C	0.401748	-2.4062	-0.42935	0.606996	-2.58499	-1.26541	0.058133	-2.23659	-1.03845	0.35016	-2.16132	1.223753	0.064092	-1.23015	2.224528
C	4.251754	0.737563	0.462465	3.619039	0.758649	1.042023	3.697127	0.434522	1.240451	4.166934	0.541159	-0.71359	3.733954	-0.19257	-1.11414
C	-1.33071	0.087617	-0.7031	-1.51927	-0.49504	-1.19882	-1.36098	0.423226	-1.47402	-1.37331	0.299645	0.766217	-1.43126	1.116341	1.023974
C	-2.16537	0.313389	0.560613	-2.57612	0.432181	-0.61131	-2.43788	1.130746	-0.64915	-2.18202	0.614828	-0.49523	-2.41294	1.246323	-0.1439
C	-3.64037	0.381868	0.262236	-2.69736	0.273476	0.882074	-2.89571	0.307974	0.527215	-3.65735	0.707156	-0.20821	-2.75179	-0.08923	-0.75588
O	-4.3676	0.503232	1.402092	-3.46721	1.249622	1.424864	-3.64715	1.044403	1.384468	-4.36561	0.932776	-1.34417	-3.40066	0.058559	-1.93852
O	-4.14098	0.33963	-0.83588	-2.20091	-0.61212	1.537708	-2.65745	-0.86319	0.705036	-4.17444	0.601321	0.878194	-2.50964	-1.16886	-0.27097
H	2.970816	0.895797	-1.26924	2.586816	1.19149	-0.80445	2.950774	0.902612	-0.73123	2.270252	0.264714	-1.70862	1.667346	-0.53212	-1.64373
H	2.107079	2.544234	0.399749	1.18701	2.159605	1.021785	1.75282	2.419882	0.8552	2.157694	2.492456	-0.583	1.984897	1.947352	-1.57393

H	1.730767	1.19185	1.50967	0.937206	0.513066	1.682291	1.011342	0.98657	1.630271	2.319688	1.67882	1.001817	2.351885	1.828978	0.173238
H	-0.61278	-2.50822	-0.03645	-0.11437	-3.14357	-0.65755	0.089607	-2.35591	-2.12849	-0.12966	-1.99077	2.191977	-0.16197	-0.62588	3.109025
H	0.402299	-2.74518	-1.47264	0.228696	-2.55599	-2.29386	0.670323	-3.03239	-0.60921	-0.37135	-2.66755	0.571344	-0.84768	-1.75892	1.924834
H	1.058266	-3.08345	0.122045	1.550423	-3.13463	-1.26558	-0.97347	-2.36357	-0.70025	1.192125	-2.84081	1.370472	0.815006	-1.97297	2.500519
H	4.966681	0.052628	0.003158	4.513242	0.3502	0.567962	3.328074	0.063833	2.203114	4.562835	0.678588	0.298655	4.02911	-1.24199	-1.16626
H	4.185319	0.479635	1.525218	3.458613	0.206003	1.974223	4.142498	1.422825	1.395188	4.450356	1.408357	-1.31896	4.322192	0.274103	-0.31627
H	4.631217	1.760853	0.375555	3.794143	1.811549	1.28664	4.470971	-0.25521	0.899074	4.635826	-0.35187	-1.13034	3.983124	0.295654	-2.06213
H	-1.53825	0.893891	-1.4157	-1.48833	-0.36501	-2.28974	-1.16139	1.029749	-2.36832	-1.7213	-0.63853	1.200536	-1.82789	0.418863	1.763089
H	-1.61806	-0.84894	-1.18428	-1.79696	-1.53828	-1.01967	-1.71927	-0.55078	-1.81032	-1.54943	1.087293	1.509309	-1.33206	2.100424	1.50152
H	-1.99775	-0.48071	1.29924	-2.3752	1.483796	-0.83549	-2.09404	2.105583	-0.28999	-1.86107	1.562215	-0.94384	-3.35572	1.690106	0.202096
H	-1.87482	1.249606	1.053717	-3.55841	0.205912	-1.04614	-3.32344	1.326203	-1.26826	-2.02783	-0.15046	-1.26678	-2.02935	1.91062	-0.92426
H	-5.29911	0.548438	1.133489	-3.5193	1.06461	2.376102	-3.9275	0.443937	2.093631	-5.29898	0.983936	-1.08334	-3.61028	-0.83553	-2.2528

Table S7. The coordinates for the lowest energy conformers of **14b**.

No.	conformer 1			conformer 2			conformer 3			conformer 4			conformer 5		
C	2.914017	-0.54818	-0.13432	2.418151	-0.60865	0.119782	2.580395	-0.48249	0.195166	2.712121	-0.71218	-0.26013	2.1369	-0.48754	0.71557
C	1.891463	-1.44172	0.535753	1.142894	-1.09776	0.772553	1.465066	-1.37538	0.693851	2.000833	-1.18781	0.988474	1.082006	0.021746	1.673985
O	0.615795	-1.39446	-0.13474	0.003713	-0.94995	-0.10079	0.383906	-1.45839	-0.25541	0.571253	-1.03643	0.884177	-0.21812	0.105488	1.05376
C	0.143279	-0.15212	-0.39718	-0.13816	0.278289	-0.64495	-0.06736	-0.26833	-0.72358	0.143518	0.176159	0.459388	-0.2455	0.698272	-0.16002
C	0.8975	0.978107	-0.32858	0.854192	1.206865	-0.70797	0.607782	0.906054	-0.60064	0.9434	1.104162	-0.13438	0.85912	0.976666	-0.90258
C	2.350022	0.871522	-0.19034	2.216324	0.840049	-0.33018	1.992732	0.893082	-0.12546	2.30461	0.736568	-0.53123	2.151989	0.42146	-0.51438
O	3.08475	1.851205	-0.18963	3.166068	1.608885	-0.42233	2.678424	1.904865	-0.04847	3.05804	1.506018	-1.11402	3.176586	0.596458	-1.16285
C	0.32583	2.356277	-0.51899	0.611267	2.583752	-1.26402	0.027102	2.224091	-1.03728	0.447537	2.474572	-0.51785	0.768742	1.721988	-2.20608
C	4.26348	-0.60797	0.570485	3.616502	-0.76308	1.04858	3.728064	-0.40286	1.194168	4.222278	-0.87651	-0.14367	3.501869	-0.58255	1.385223
C	-1.30914	-0.21246	-0.74946	-1.51559	0.493173	-1.20532	-1.37702	-0.4488	-1.43016	-1.32368	0.33038	0.70451	-1.64679	1.0345	-0.58546

C	-2.17208	-0.39835	0.501375	-2.57552	-0.43012	-0.61689	-2.43116	-1.16748	-0.58591	-2.12612	-0.67595	-0.12563	-2.71385	0.149165	0.050543
C	-3.63539	-0.528	0.167187	-2.70023	-0.26563	0.875555	-2.87141	-0.35133	0.601784	-3.60782	-0.54449	0.10907	-2.53758	-1.30148	-0.31819
O	-4.38776	-0.64685	1.29083	-3.47491	-1.23711	1.41983	-3.6055	-1.09386	1.468587	-4.30637	-1.4176	-0.66072	-3.34933	-2.10251	0.416924
O	-4.10797	-0.52959	-0.94406	-2.20243	0.620573	1.529363	-2.63435	0.820018	0.779902	-4.1368	0.225365	0.874697	-1.792	-1.72422	-1.1694
H	3.028982	-0.87493	-1.18144	2.586919	-1.19354	-0.79994	2.950859	-0.88814	-0.76128	2.341099	-1.29853	-1.11754	1.828777	-1.48292	0.354641
H	1.735534	-1.11801	1.578201	0.932879	-0.51485	1.683683	1.054792	-0.9743	1.635255	2.336075	-0.6009	1.859296	1.347874	1.032646	2.024058
H	2.187893	-2.49439	0.527769	1.183334	-2.16155	1.023735	1.794246	-2.40596	0.855196	2.170051	-2.25001	1.186421	0.95082	-0.63489	2.538807
H	0.393711	2.680695	-1.56463	0.240964	2.555522	-2.2954	0.026084	2.331133	-2.12901	-0.01951	2.993972	0.324343	0.130205	2.607723	-2.13352
H	-0.71856	2.422846	-0.20539	-0.11544	3.140347	-0.66079	-0.99522	2.349756	-0.67113	1.298247	3.067432	-0.85984	1.771963	2.038294	-2.49971
H	0.911431	3.068097	0.068009	1.553954	3.1346	-1.25635	0.646971	3.027625	-0.63431	-0.27941	2.436407	-1.33782	0.377281	1.084836	-3.00951
H	4.96277	0.086896	0.10203	3.454786	-0.21169	1.98131	3.373338	-0.02879	2.160905	4.71327	-0.47799	-1.03315	3.485503	-1.30365	2.209051
H	4.164058	-0.31528	1.621618	3.790688	-1.81641	1.291981	4.183464	-1.38686	1.347117	4.605288	-0.32132	0.719663	4.254802	-0.89056	0.657651
H	4.683035	-1.6184	0.528742	4.511766	-0.35438	0.576721	4.490665	0.290396	0.835057	4.494217	-1.93086	-0.02841	3.807641	0.392442	1.780703
H	-1.61485	0.684999	-1.29016	-1.79259	1.537535	-1.03212	-1.75187	0.521411	-1.75937	-1.53404	0.164743	1.768035	-1.85157	2.081576	-0.31961
H	-1.4676	-1.06339	-1.42148	-1.48139	0.357456	-2.29547	-1.18766	-1.05285	-2.32838	-1.64527	1.344643	0.46374	-1.70644	0.96526	-1.6751
H	-1.86721	-1.2956	1.054443	-3.55635	-0.20392	-1.05499	-3.32719	-1.36882	-1.18776	-1.93446	-0.54587	-1.19853	-2.72611	0.23479	1.140924
H	-2.05161	0.440284	1.199153	-2.37554	-1.48286	-0.83658	-2.07212	-2.14025	-0.23614	-1.83178	-1.70557	0.109423	-3.70955	0.456203	-0.29461
H	-5.31009	-0.72847	1.000033	-3.52852	-1.04859	2.370298	-3.87541	-0.4976	2.185305	-5.24483	-1.27824	-0.45652	-3.19736	-3.00954	0.107095

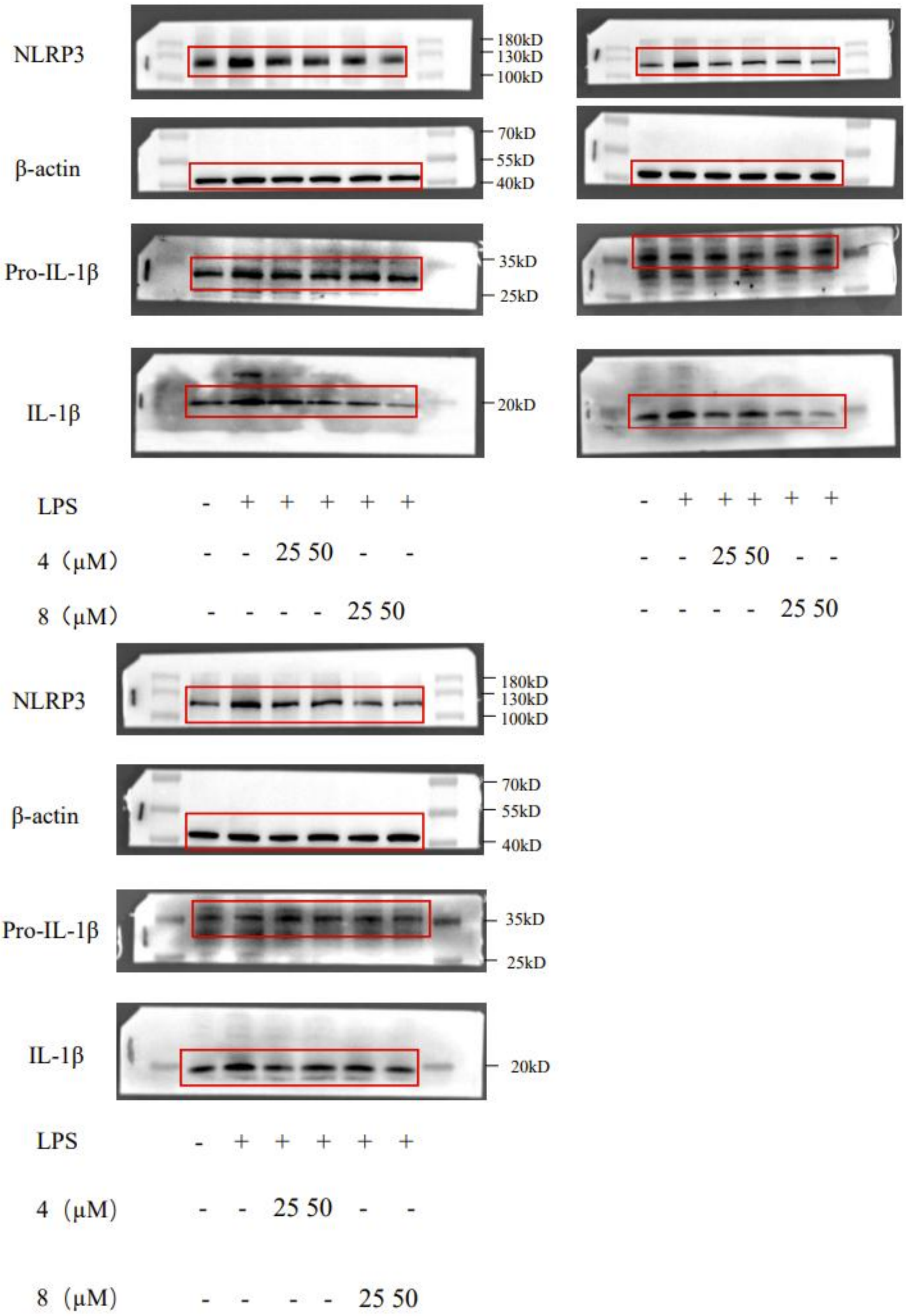

Figure S36. Uncropped images of gel/blot

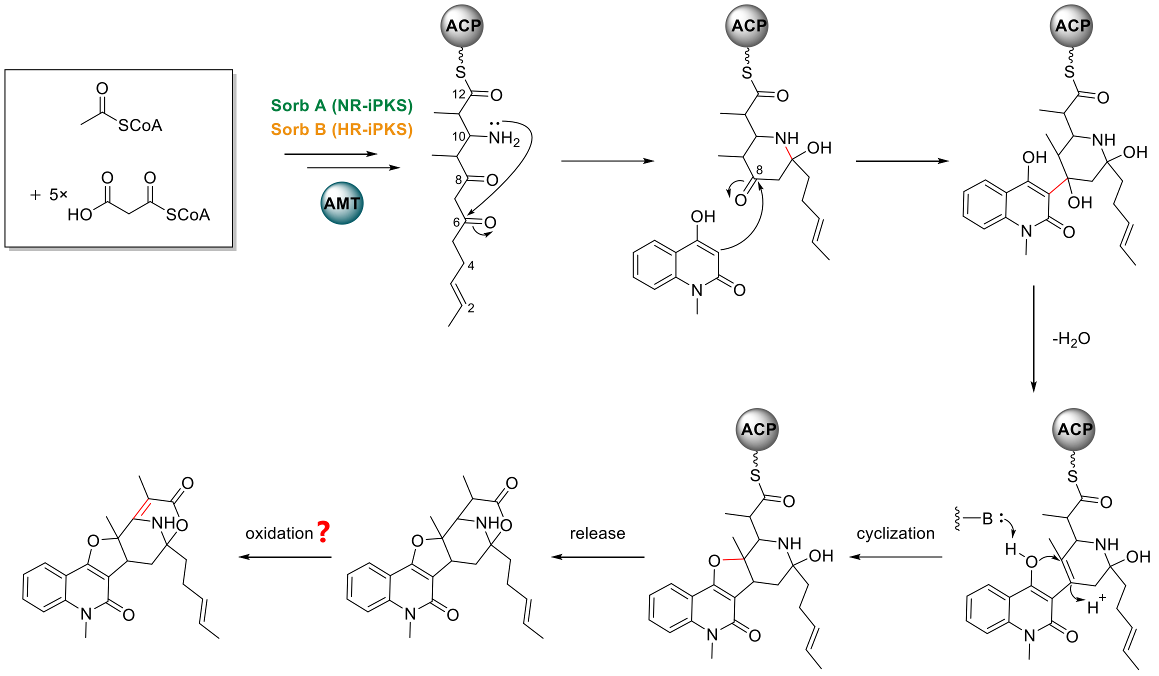

Scheme S1. Proposed biosynthetic pathway of quinosorbicillinol (**1**).
